# The exponential distance rule-based network model predicts topology and reveals functionally relevant properties of the *Drosophila* projectome

**DOI:** 10.1162/netn_a_00455

**Published:** 2025-07-29

**Authors:** Balázs Péntek, Mária Ercsey-Ravasz

**Affiliations:** Faculty of Physics, Babeş-Bolyai University, Cluj-Napoca, Romania; Transylvanian Institute of Neuroscience, Cluj-Napoca, Romania

**Keywords:** Structural brain networks, *Drosophila* connectome, Neuropil network, Exponential distance rule, Asymmetric connections, Functional hierarchy

## Abstract

Studying structural brain networks has witnessed significant advancement in recent decades. Findings revealed a geometric principle, the exponential distance rule (EDR) showing that the number of neurons decreases exponentially with the length of their axons. This neuron-level information was used to build a region-level EDR network model that was able to explain various characteristics of interareal cortical networks in macaques, mice, and rats. The complete connectome of the *Drosophila* has recently been mapped providing information also about the network of neuropils (projectome). A recent study demonstrated the presence of the EDR in the *Drosophila*. In our study, we first revisit the EDR itself and precisely measure the characteristic decay rate. Next, we demonstrate that the EDR model effectively accounts for numerous binary and weighted properties of the projectome. Our study illustrates that the EDR model is a suitable null model for analyzing networks of brain regions, as it captures properties of region-level networks in very different species. The importance of the null model lies in its ability to facilitate the identification of functionally significant features not caused by inevitable geometric constraints, as we illustrate with the pronounced asymmetry of connection weights important for functional hierarchy.

## Author Summary

Recent advancements in the structural brain network analysis have revealed the exponential distance rule (EDR), which shows that neuron numbers decrease exponentially with their axon length. This neuron-level insight led to a region-level EDR network model that explained properties of interareal cortical networks in macaques, mice, and rats. Recently, the complete connectome of the *Drosophila* has been mapped and the presence of the EDR has also been confirmed. Our research revisits the EDR, measures its decay rate, and demonstrates that the EDR model effectively accounts for various properties of the network of brain regions, serving as an appropriate null model for a structural brain network analysis. Its importance as a null model lies in its ability to facilitate the identification of functionally significant features not caused by inevitable geometric constraints.

## INTRODUCTION

In experimental and theoretical studies, much attention has been paid in the last few decades to understanding the properties of the complex structural network of the brain, because these are key to its functioning. Experiments have been focusing mainly on mapping these structural brain networks in different species. In mammals, the mapping is usually performed on meso- or macroscale: finding the connections between functional areas. Great advances have been made with retrograde tracing experiments in macaques (Markov et al., 2011, 2014) and anterograde and retrograde tracing in mice (Gămănuţ et al., 2018; Horvát et al., 2016; Oh et al., 2014; Zingg et al., 2014). In humans, only noninvasive techniques (e.g., diffusion tensor imaging) are available; however, these methods are indirect and can have issues with false-positive and false-negative connections (de Reus & van den Heuvel, 2013; Donahue et al., 2016; Dyrby et al., 2007; Knösche et al., 2015; Maier-Hein et al., 2017; Thomas et al., 2014). Mapping the brain on neuronal level has started with *C. Elegans* (White et al., 1986), and recently, the whole brain of the *Drosophila* fruit fly has been mapped (Buhmann et al., 2021; Dorkenwald et al., 2024; Heinrich et al., 2018; Schlegel et al., 2024; Zheng et al., 2018), the database containing a great amount of information about the approximately 140,000 neurons and 150 million synapses included in 78 neuropils (larger brain areas defined in this map; Dorkenwald et al., 2024; Schlegel et al., 2024).

Conceptualizing interareal neuroanatomy in terms of graphs (Bullmore & Sporns, 2012; Sporns, 2000, 2011) has made the extensive toolset of graph theory (Barabási & Pósfai, 2016; Newman, 2010) highly useful for analyzing cortico-cortical graphs in mammals. Interesting features related to wiring optimization have been identified, such as the small-world topology (Bassett & Bullmore, 2017; Watts & Strogatz, 1998), which balances local specialization and global integration. Additionally, hierarchical modularity has been discovered (Bullmore & Sporns, 2012; Hilgetag et al., 2000; Hilgetag & Kaiser, 2004; Meunier et al., 2010; Sporns & Betzel, 2016; van den Heuvel et al., 2012) indicating that cortical networks are organized into modules with dense intramodular connections and sparser intermodular connections, facilitating efficient information processing and communication. Later, based on the empirical observation of the exponential distance rule (EDR) in the macaque brain, a predictive network model has been defined (Ercsey-Ravasz et al., 2013), which explained many topological features of these networks, also providing deeper explanations to wiring optimization, modular structure, and efficient information processing.

The EDR states that the probability of axons with a given length decreases exponentially as function of their length: *p*(*d*) = *c* · exp(−*λ* · *d*). Simply saying, there are many neurons with short axons and only a few with long axons, this decay being exponential. This was first observed in the white matter of the macaque (Ercsey-Ravasz et al., 2013), later in the mouse (Gămănuţ et al., 2018; Horvát et al., 2016). It was shown to be true also in the gray matter of the macaque and mouse (Horvát et al., 2016) with different decay rates. A recent study suggests it also holds in marmosets, humans, and *Drosophila* (Józsa et al., 2024). It appears to be a basic geometrical principle that achieves the balance between wiring optimization and efficient communication. The *λ* decay rate is different in species depending on brain sizes.

Recent theoretical studies (Pósfai et al., 2024) also demonstrate that in physical networks where nodes and links are placed in geometrical space, have physical size, and cannot intersect, in a densely packed state, the exponential distribution of link lengths follows naturally from these geometrical constraints. These theoretical results make us expect the EDR—as observed before in the macaque and mouse—to be true probably in any brain.

While the EDR concerns the number of individual neurons as a function of axon length, providing information at the neuronal level, a one-parameter predictive region-level network model was built based on this principle (Ercsey-Ravasz et al., 2013) and explained many important properties of the interareal cortical network in the macaque and mouse (Gămănuţ et al., 2018; Horvát et al., 2016). In rats, the model was tested on two different (but still regional) scales (Noori et al., 2017), appearing to be valid only on a larger scale. However, this data set was a large collection based on the bibliography of the last 50 years (Noori et al., 2017), and information is still missing, so the validity of the model on the lower scale remains an open question.

The new experiments presented in the FlyWire database mapped the whole connectome of the *Drosophila* on a neuronal level (Buhmann et al., 2021; Dorkenwald et al., 2024; Heinrich et al., 2018; Schlegel et al., 2024; Zheng et al., 2018). Based on the length distribution of neurons in the central brain, the presence of the EDR has already been shown (Józsa et al., 2024). However, in that study, the decay rate *λ* has not been measured for the *Drosophila*. The main goal of our study is to check the validity of the region-level EDR network model (previously applied in mammals) in the *Drosophila*. For this, we first revisit the EDR itself by precisely measuring the decay rate *λ_d_* in the whole *Drosophila* brain. The FlyWire database allowed us to extract a corresponding graph for approximately 140.000 neurons, on which we were able to measure the cable length between the dendritic arbor (postsynaptic points) and axonal arbor (presynaptic points), the distributions clearly indicating the presence of EDR. Having the coordinates of all pre- and postsynaptic points, we also calculate Euclidean distances, showing how strongly they correlate with each other and what is the scaling factor between them. This “Euclidean cable length” and the corresponding decay rate *λ_ED_* is needed because we are able to construct the region-level EDR model only by using the Euclidean distances available between areas (neuropils). We build the network of neuropils using the method introduced by Dorkenwald et al. (2024; Lin et al., 2024). Neuropils also have their coordinates in the JFRC2 Template Brain dataset allowing us to build the Euclidean distance matrix between neuropils (Jenett et al., 2012). Using this distance matrix, we apply the one-parameter EDR network model first introduced by Ercsey-Ravasz et al. (2013) at different *λ* parameter values and compare its properties to the real dataset. We show that the optimal parameter reproducing a whole range of network properties coincides well with the *λ_ED_* decay rate measured directly from the EDR of Euclidean axon lengths.

The success of this region-level EDR model across species from various evolutionary branches (insects, rodents, primates) suggests that it is the appropriate null model for comparing region-level structural brain networks in future studies. The significance of the null model stems from the need to compare experimental data with models that incorporate unavoidable geometric features present in physical networks, such as the EDR model. This kind of comparisons can more easily reveal functionally relevant features that are not directly attributable to geometric factors. As we will demonstrate, a compelling illustration of these unique properties, not attributable to simple geometric factors, is the pronounced asymmetry in link weights between bidirectionally connected area pairs. This asymmetry plays a crucial role in determining the functional hierarchy of brain areas.

## RESULTS

### The *Drosophila* Database

The fruit fly (*Drosophila melanogaster*) has been used as a model organism in biology since the early 20th century. From a neuroscience standpoint, it has become an ideal experimental subject due to its rich functionality. In addition to basic functions like vision, flight, and walking, the fruit fly exhibits more complex behaviors such as courtship and aggression (Coen et al., 2014; DasGupta et al., 2014; Dorkenwald et al., 2022; Duistermars et al., 2018; Jennings, 2011; Owald et al., 2015; Seelig & Jayaraman, 2015).

In 2018, a breakthrough occurred in the field of connectomics when Zheng et al. (2018) successfully mapped the entire brain of the fruit fly. Researchers developed a serial section transmission electron microscope (ssTEM) that allowed for the mapping of the entire adult fruit fly brain at the synaptic level, with nanometer resolution (see details in the Materials and Methods section). In 2021, Julia Buhmann et al. (2021) succeeded in training a convolutional neural network with tens of millions of parameters capable of recognizing pre- and postsynaptic points without the need for reconstructed neurons. Their method allows for determining whether a voxel is a postsynaptic side and, if so, calculating a vector pointing to the presynaptic side. Within the connectome, the authors claim that connections within the network can be determined with approximately 95% accuracy by filtering out connections with fewer than five synapses (Buhmann et al., 2021).

The FlyWire project was launched around the beginning of 2022 (Dorkenwald et al., 2022). As described in the Materials and Methods section, we downloaded the data from the Codex platform (the version of the database: snapshot 783—Oct 2023), which contains 139,255 validated neurons, 2,700,513 thresholded connections with 34,153,566 synapses. The database administrators specifically noted that the 2.7 million connections between neurons represent a subset of all detections, and these connections contain more than four synapses. This threshold value coincides with the value introduced by Buhmann and colleagues for synapse prediction. In addition to predicting these filtered connections with very high accuracy, the use of the threshold value can also be argued from a biological perspective. Stronger (multisynaptic) connections may play a more important role in communication between neurons, and it is more likely that these are independent of individual differences (Dorkenwald et al., 2024). We exclusively analyzed intrinsic neurons, totaling around 118,000 (85%), excluding afferent and efferent neurons that may have different structures due to their roles in sending or receiving information outside the brain (Dorkenwald et al., 2024).

### The EDR in *Drosophila*

**Figure 1.**
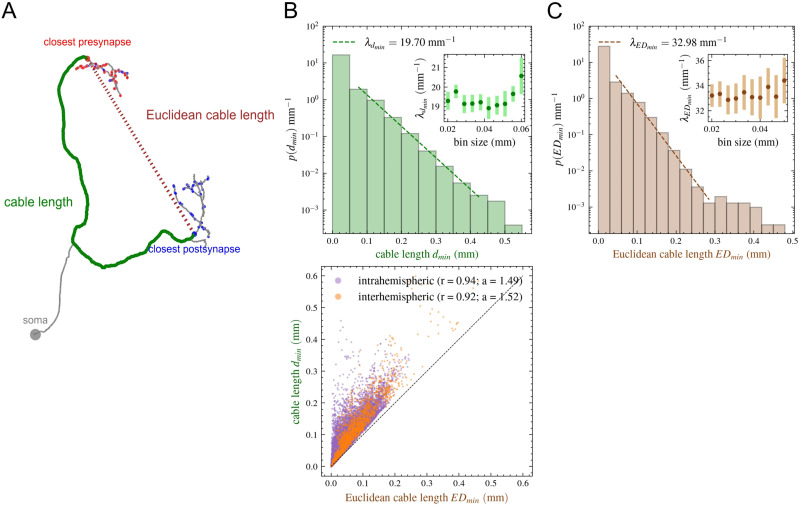
Measuring cable length. (A) Tree of a neuron with presynaptic (red) points and postsynaptic (blue) points identified. The green path shows the main cable length, the brown line indicates the Euclidean cable length. (B) Probability distribution of cable lengths showing the EDR. Using different bin sizes and taking into consideration the uncertainty resulting from the fitting, we estimate *λ*_*d*_*min*__ = [18.4, 21.5] mm^−1^. (C) EDR of Euclidean cable lengths providing *λ*_*ED*_*min*__ = [31.4, 36.2] mm^−1^. (D) Correlation plot for cable length and Euclidean cable length. Neurons were separated based on the location of their closest post- and presynaptic point to the soma: Those on the same sides (left, right, center) were considered intrahemispheric while the rest were interhemispheric. Both groups exhibit a strong correlation of *r* = 0.9, and a scaling factor of *a* = 1.5. The dashed black line represents the line of equality.

In order to measure the decay rate of the EDR, we needed to measure the cable lengths. While a recent study (Józsa et al., 2024) has shown the presence of the EDR in the *Drosophila*’s central brain, they did not provide an exact value for the decay rate *λ*. In their study, the cable length values provided in the database were used, which is defined as the total sum of distances between neighboring nodes in the neuron tree (including the whole tree structure of the axon and dendrite). This can result in very long cable lengths, and while the exponential nature of those length values already indicates the presence of EDR, here, we needed to precisely determine the *λ_d_* decay rate corresponding to the main cable lengths responsible for information transfer. In the fruit fly, neurons have a special structure, different from the structure of neurons in brains of vertebrates. The soma is in a layer on the periphery of the brain, disjoint both from the dendritic and axonal arbor. Starting from the soma, a primary neurite penetrates into the interior of the brain, where it then branches into two parts: dendrite and axon (e.g., see Figure 1A). The main transmission cable is considered to be between the dendritic and axonal arbor. Therefore, we downloaded spatial graph/tree structures from the Codex web interface (Matsliah et al., 2023), which correspond to the “skeleton” of real neurons (their digital representation). Using these neuron trees, we measured the cable lengths as follows: (a) We determined all voxels indicating the presynaptic points of that neuron (synapses between neurons with connections below the five-synapse threshold are not considered); (b) given one presynaptic point, we measured the shortest path between the soma and the given point on the tree structure of the neuron downloaded from the database; (c) we calculated these distances for all presynaptic points of a neuron, we identified the closest presynaptic point (with minimal distance), and we also calculated the average for all presynaptic points. (d) We repeated the first three steps for all postsynaptic points, identified the postsynaptic point closest to the soma, and calculated the average path for all postsynaptic points. (e) The goal through these steps is to identify the closest presynaptic point on the axonal arbor and the closest postsynaptic point on the dendritic arbor. Once these are identified, the shortest path is calculated between these two points providing the length of the main transmission cable (in this shortest path, the soma and the primary neurite are not considered anymore); see Figure 1A.

We select the nearest pre-/postsynaptic points to the cell body because we are interested in the main cable length itself. After the closest pre-/postsynaptic point, the axon/dendrite almost always branches into many directions (Figure 1A). It should also be mentioned that for certain neurons, the nearest presynaptic point is not necessarily located around the end of the axon, but it may be much closer on the axon or even on the dendrite. The same is true for postsynaptic points that can be sometimes much closer to the soma and then the dendritic arbor itself. There are two possible reasons for this: The synapse-predicting neural network makes errors, or these points may represent nontraditional synapses, such as dendrite–dendrite or axon–axon connections (Dorkenwald et al., 2024; Eckstein et al., 2024; Galindo et al., 2023; Meinertzhagen, 2018; Schneider-Mizell et al., 2016; Winding et al., 2023; Supporting Information Figure S1A). To eliminate these errors and special cases, we calculate the difference between the path length of the closest presynaptic (postsynaptic) point and the average path length to all presynaptic (postsynaptic) points of that neuron. The distributions of these differences are shown in Supporting Information Figure S1B for all neurons. We use a threshold of 0.15 mm (for both type of synapses) to eliminate neurons where errors are most probable, and in Figure 1B and Figure 1C, we plot the EDR for the remaining 103,179 neurons (only 11% of neurons are eliminated from the statistics). The EDR distributions using different thresholds (0.075, 0.1, 0.15, and 0.2 mm and no threshold) are shown in Supporting Information Figure S1C and Figure S1D. We can see that the difference is not large; nevertheless, the EDR is clearer when eliminating the errors.

Figure 1B shows the probability distribution of cable lengths for these neurons. The histogram shows the probability of a neuron having a given cable length. Setting the *y* axes on log scale, we can indeed observe the presence of the exponential decay. Changing the bin size may slightly change the fitted parameter of the exponential distribution, which can be approximated in the *λ_d_min__* = [18.4, 21.5] mm^−1^ interval.

The distances between neuropils are Euclidean distances calculated based on their coordinates. When later building the EDR-based random networks, we will need to estimate the probabilities of neurons projecting to certain Euclidean distances. For this reason, after calculating the cable length based on the shortest path calculated from the skeleton of the neuron, we also calculate Euclidean distances between the closest post- and presynaptic points (see Figure 1A). In Figure 1C, we plot the EDR for the Euclidean cable lengths measured. In this case, we obtain an interval of *λ_ED_min__* = [31.4, 36.2] mm^−1^ for the minimum length. In the following sections, we will use many times the *λ_ED_min__* ≅ 33 mm^−1^ when comparing the dataset with the model, because, as explained in the next section in the model, we can use only the Euclidean distances between areas; so estimating the number of neurons projecting between areas, we have to use the Euclidean cable lengths and the corresponding EDR decay parameter.

As expected, there is a strong correlation between the cable lengths itself (the shortest path on the skeleton) and Euclidean cable lengths, identifying a relatively large scaling factor of approximately 1.5 (Figure 1D). In Figure 1D, we colored separately the dots corresponding to intra- and interhemispheric projections. For the intrahemispheric group, neurons considered had their closest post- and presynaptic points on the same side (either left, right, or center); all the rest were considered to cross hemispheres. We can see that interhemispheric projections are generally longer, but strong deviations between the cable length and the Euclidean distance appear both in intra- and interhemispheric projections. This strong deviation is an important difference compared with mammals. Because of the small brain size of the fruit fly, the neuronal cables can be extremely long compared with the distances between neuropils identified. This difference is not so significant in the larger brains of macaques or even mice, where one can more easily estimate and measure the paths of axon bundles going through the white matter between functional areas. In the fruit fly, each neuron has an independently complex tree structure, and projecting cables do not necessarily travel through the brain in a direct, relatively straight path.

### The Network of Neuropils in the *Drosophila*

The brain of the fruit fly is much denser (6.9 synapses/*μ*m^3^) than that of mammals (< 1 synapse/*μ*m^3^), and the structure of most of its neurons differs from what is typical in mammals, in the sense that the soma and dendrites are most of the time spatially separated (Dorkenwald et al., 2024). The soma are mainly located on the surface of the brain, with a primary neurite penetrating into the interior of the brain, where it then branches into two parts: dendrite and axon (e.g., see Figure 1A). Therefore, in the case of the fruit fly, associating a single neuron with a single brain region is not possible.

**Figure 2.**
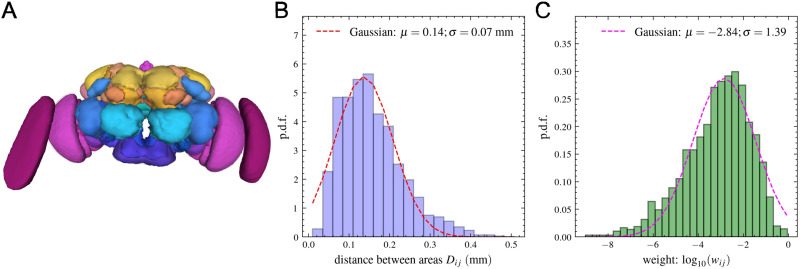
The neuropil network. (A) The atlas of neuropils, image obtained from the *Connectome Data Explorer* (*Codex*) web-app (codex.flywire.ai/app/neuropils). (B) Distribution of Euclidean distances between neuropils. (C) Distribution of logarithmic weight values (log_10_*w*_*ij*_) is a normal distribution, indicating that the weight distribution is lognormal.

Although neurons cannot be associated, it is possible to associate synapses to brain regions. This was successfully achieved by Dorkenwald and colleagues, utilizing a previously determined atlas of neuropils (brain regions) from a prior study (Ito et al., 2014), categorizing synapses into 78 zones based on their presynaptic sides. The same team even developed a method of defining the projectome on the level of neuropils (Dorkenwald et al., 2024; Lin et al., 2024). We used the same method, as described shortly in the Materials and Methods section. The matrix elements obtained actually represent a strength value, characterizing the connections between neuropils (brain regions). Considering that brain regions can vary in size (see Figure 2A), the numbers of synapses can differ significantly between larger and smaller neuropils. Therefore, we normalized the obtained connectivity matrix by columns, so that the newly obtained *w*_*ij*_ matrix element will correspond to the probability of information from area *i* flowing to area *j*. This way it has similar meaning to the fraction of labeled neurons applied in retrograde tracing studies in macaques and mice (Ercsey-Ravasz et al., 2013; Horvát et al., 2016), where also neuron counts are normalized separately for each injection.

To calculate the distances between brain regions, we used the locations of their center of mass published in an earlier study (Jenett et al., 2012), obtained through the fafbseg.py Python package. These coordinates were available for a total of 75 brain regions, as the FlyWire team added three brain regions to the database later on. For our purposes, this did not represent a significant loss, as these three zones are already close to sensory organs, and we are mainly interested in intrabrain connections (analyzing only intrinsic neurons), as mentioned above. Two of these zones are associated with the left and right eyes (lamina of the compound eyes—see the two outer magenta zones in Figure 2A), while the third is the ocellar ganglion located on top of the head (see the small pink sphere at the top in Figure 2A) playing a role in flight and spatial orientation. Therefore, we recalculated the projectome only for these *N* = 75 brain regions (neuropils), resulting in a network with M = 4,733 connections (density: 85%).

In Figure 2B, we plot the distribution of Euclidean distances between neuropils, which can be well approximated with a truncated Gaussian distribution. This is somehow expected from the spatial positioning of neuropils. The distribution of normalized connection weights (*w*_*ij*_) shows a lognormal distribution ranging over several orders of magnitudes. As first explained in the case of the macaque, the Gaussian distribution of distances combined with the EDR gives a theoretical support for the lognormal distribution of connection weights (Ercsey-Ravasz et al., 2013; Horvát et al., 2016). As we can see, this is also the case for the fruit fly.

**Figure 3.**
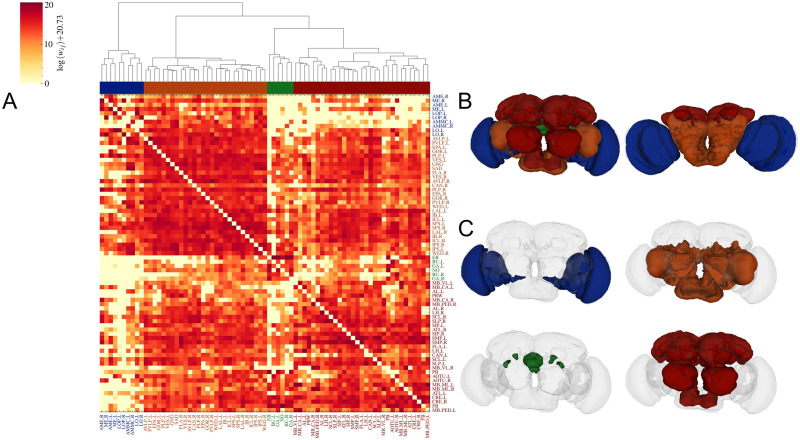
Modular structure of the *Drosophila* neuropil network. (A) The weighted connectivity matrix with the areas ordered according to the dendrogram provided by the hierarchical clustering based on Ward’s method (see the Materials and Methods section). We color the four largest clusters under the dendrogram. (B) The four largest clusters presented together on the *Drosophila* brain from a front and a back view. (C) The four clusters are presented separately from the front view. The blue cluster contains mainly areas from the optic lobe. The orange cluster contains all ventromedial regions, almost the whole ventrolateral part, some parts of the lateral complex and of the inferior protocerebrum, the gnathal ganglia, and two right-side periesophageal regions (FLA_R, CAN_R). The green cluster is concentrated around the central complex (EB and NO) including also parts of the lateral complex. The red cluster includes the whole superior protocerebrum, the mushroom body, the antenna lobe, the lateral horn, some regions from the inferior protocerebrum, the ventrolateral part, the central complex, and the periesophageal areas. The six and eight largest clusters are shown in Supporting Information Figure S2.

We first investigated the modularity of the network with a hierarchical clustering method (Murtagh & Legendre, 2014; Ward, 1963; see the Materials and Methods section); the weighted connectivity matrix with the dendrogram and the four largest modules colored are shown in Figure 3. The clustering provides a realistic modular structure with symmetric organization and clusters localized in space.

The first big cluster (blue; Figures 3B and 3C) contains mainly the left and right optic lobe with the medulla (ME), accessory ME (AME), lobula (LO), and LO plate (LOP) and interestingly also includes the antennal mechanosensory and motor center (AMMC) from the periesophageal regions.

The second cluster (orange; Figures 3B and 3C) is located mainly at the inferior and back side of the brain. It includes the lateral accessory lobe (LAL); the inferior clamp (ICL) and inferior bridge (IB) from the inferior protocerebrum; all ventromedial regions: vest (VES), superior posterior slope (SPS), inferior posterior slope (IPS), epaulette (EPA), and gorget (GOR); almost the whole ventrolateral part: anterior and posterior ventrolateral protocerebrum (AVLP, PVLP), posteriolateral protocerebrum (PLP), and wedge (WED); the gnathal ganglia (GNG); and interestingly two right-side periesophageal regions: right flange (FLA_R) and cantle (CAN_R).

The third cluster (green; Figures 3B and 3C) contains a large part of the central complex: the ellipsoid body (EB) and Noduli (NO); and surprisingly even if they are not neighbors in space, it also includes the left and right bulb (BU) and gall (GA) from the lateral complex, meaning there are strong connections between them.

The fourth cluster (red; Figures 3B and 3C) is localized more at the top and frontal side of the brain. It contains the whole superior protocerebrum: medial (SMP), intermediate (SIP), and lateral (SLP); the rest of the inferior protocerebrum: antler (ATL), crepine (CRE), and superior clamp (SCL); one single region from the ventrolateral part: the anterior optic tubercle (AOTU); the whole mushroom body with the vertical and medial lobe (MB_VL, MB_ML), pedunculus (MB_PED), and calyx (MB_CA); the antenna lobe (AL); the rest of the central complex: the fan body (FB) and protocerebral bridge (PB); the lateral horn (LH); from the periesophageal regions the prow (PRW); and only the left cantle (CAN_L) and left flange (FLA_L). These two regions (CAN and FLA) are the only ones where the left and right parts are not in the same cluster.

In Supporting Information Figure S2, we show the six and eight largest clusters, respectively, where the clusters remain mostly symmetric and show realistic spatial distribution. Interestingly, the blue cluster containing the optical lobes gets divided into subgroups of the MEs, LOs, and AMMCs, with the LOPs further being separated into a single cluster. Some asymmetry is introduced into the spatial structure from the hemispheres of the lateral horn, antler, and also certain parts of the mushroom body.

We also performed the hierarchical clustering with other combinations of cluster linkages and node similarity metrics (see the Materials and Methods section). One example would be the average linkage paired with correlation similarity (Supporting Information Figure S3), for which the four largest clusters share a high resemblance with those obtained with Ward’s algorithm. Notable differences include the separation of AMMC and LOP from the other optic lobes; the lateral complex fully joining the central (EB and NO); and the left and right parts of the LO grouped into different clusters. Another method pairs the complete cluster linkage with a cosine node similarity; the result is shown in Supporting Information Figure S4.

### The EDR-Based Network Model

The EDR-based network model is a one-parameter maximum entropy-based model (Jaynes, 1957) first introduced by Ercsey-Ravasz et al. (2013). The goal is that knowing the distance matrix between brain areas *D*_*ij*_ and the number of connections *M* in the structural network, the model should generate a random network with the same density, which takes into account only the presence of the EDR with a given decay parameter *λ*, but everything else is taken as random. Comparing these random networks with the real data can provide information about the properties of the network that are explained by this geometrical rule.

In our case, the input is the distance matrix between the 75 neuropils (obtained from JFRC2 Template Brain dataset; Jenett et al., 2012), *D*_*ij*_, and the number of projections in this network*M* = 4,733. The steps of generating the random network are the following: (a) We randomly choose a distance value based on the exponential distribution with a given parameter *λ*; (b) we choose uniformly at random an area pair with distance from the distance bin in the histogram of distances corresponding to the distance chosen in point 1; (c) we randomly choose the source and target from the two selected areas, and we insert a directed connection. Multiple connections between areas are allowed, generating the connection weights between areas. (d) We stop when the number of binary links between areas reaches *M*. This way, we obtain a model network with the same density as the dataset. (e) At the end, we normalize the columns of the weighted matrix (the sum of weights in each column will be 1), the weights meaning probabilities of information transfer.

For every *λ* = 0, 5, 10, 15, 20, 25, 30, 33, 35, 38, 40, 45, 50, 55, 60 mm^−1^, we repeated this procedure generating 1,000 random graphs and we measured all types of binary and weighted network properties, comparing them to the properties of the real neuropil network. In the following sections, the value of *λ* = 33 mm^−1^ will be frequently used during the comparison as this is the estimated *λ_ED_min__* decay rate resulting from the experimental data (see Figure 1C). Also, the model with *λ* = 0 mm^−1^, which we call the constant distance rule (CDR) model will be frequently used as this is the one expected to be least similar to the data set. We briefly mention here that the CDR is not simply a random model, such as an Erdos-Renyi graph (or some similar weighted version). It still follows the steps described above, taking into account the *D*_*ij*_matrix, but the final distribution of cable lengths will be constant (independent of distances).

The definitions of network properties calculated can be found in the Materials and Methods section. It is important to mention that the 1,000 model networks are all analyzed separately, the network properties being calculated for each of them individually. Only later may we apply statistics, such as calculating averages or standard deviation values. One should not consider averaging the weighted networks and building one averaged network, as this may provide a network with a different density and different properties, inducing misleading results (Varga et al., 2024).

### Comparing Binary Network Properties

**Figure 4.**
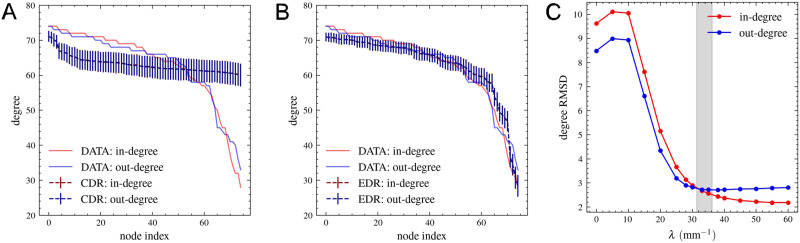
Node degrees in descending order: dataset versus model. (A) in-degree (red) and out-degree (blue) of nodes are shown for each node in decreasing order for the dataset (continuous line), CDR model with *λ* = 0 mm^−1^, and (B) EDR model with *λ* = 33 mm^−1^. For the models, dots represent the average obtained over the 1,000 generated networks; vertical lines show the standard deviation. (C) RMSD as function of *λ*, the gray interval showing the fitted values in Figure 1C: *λ*_*ED*_*min*__ = [31.4, 36.2] mm^−1^.

First, we looked at the degree distributions of nodes. Having a small network with 75 nodes, these are relatively noisy (see Supporting Information Figure S5), so here, we rather plot the in- and out-degree of nodes in decreasing order (Figures 4A and 4B) and compare these with curves generated by the CDR model with *λ* = 0 mm^−1^ and the EDR model with *λ* = 33 mm^−1^, this value being based on the fit in Figure 1C. Figure 4C shows the root-mean-square deviation (RMSD; see the Materials and Methods section) between these curves provided by the data and model as function of the *λ* parameter, indicating that the minimum differences are indeed in/very close to the ones in the *λ_ED_min__* = [31.4, 36.2] mm^−1^ (gray interval) provided by the fitting in Figure 1C (Figure 4).

**Figure 5.**
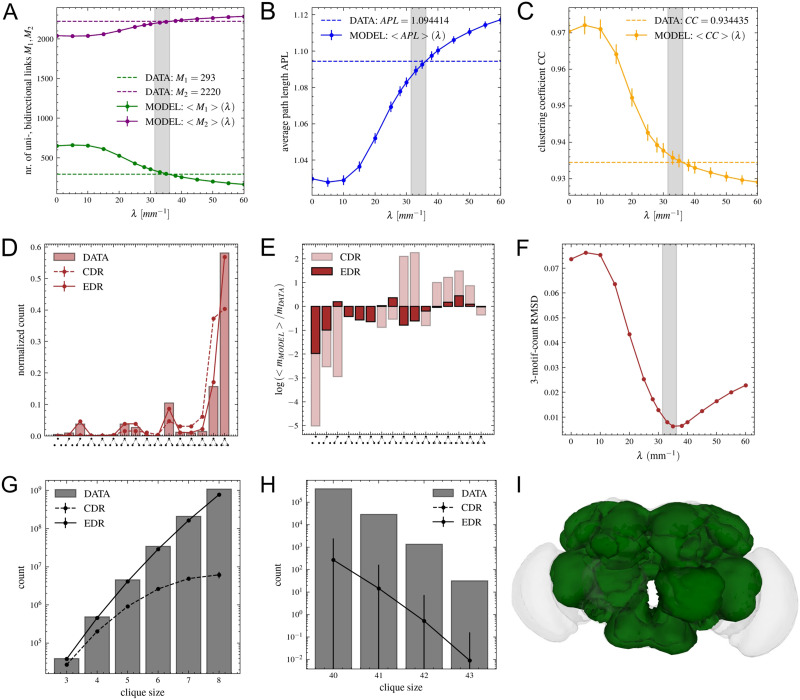
Comparing the binary properties of the neuropil network with the EDR model. (A) Number of unidirectional (green) and bidirectional (purple) links as a function of *λ*. Dashed lines show values in the neuropil network. For the model, dots represent the average obtained over the 1,000 generated networks; vertical lines show the standard deviation. Error bars are not visible on (A) because standard deviation values are very small. (B) Average binary path length as a function of *λ*. (C) Clustering coefficient as a function of *λ*. (D) Distribution of triangular motifs for data (bars), CDR (dashed line), and EDR with *λ* = 33 mm^−1^ (continuous line). (E) Relative differences between the average obtained from the 1,000 model networks and the dataset are shown on the log scale. (F) RMSD for three motif counts as a function of *λ*. (G, H) Clique size distributions shown for 3 to 8 and 40 to 43, this being the largest clique in the data. The values are shown on the log scale. The CDR cannot generate large cliques; for that reason, the curve of the CDR is not visible on H. Also for the EDR model, the number of large cliques is highly fluctuating, generating a huge standard deviation (large error bars on the log scale). (I) The total set of 53 areas included in the largest cliques is shown on the brain map (see also Supporting Information Figure S6).

Next, we compared the number of uni- and bidirectional links in the dataset and the model networks generated with different *λ* parameters. In Figure 5A, we can see how the values provided by the model agree with the data exactly in the gray interval based on the fitted *λ_ED_min__* values. Similar observations can be made for the average binary path length (Figure 5B), clustering coefficient (Figure 5C), and triangular motifs (Figure 5D–F; definitions can be found in the Materials and Methods section). For the triangular motifs, we first show the distribution of the 16 possible directed triangular motifs in Figure 5D, and then the relative differences between the data and the average provided by the network models are shown on the log scale (Figure 5E). The RMSD between the data and the model (average) distributions is shown in Figure 5F, again providing minimal values in the *λ* interval (gray) measured in Figure 1C. Because of the high network density, it is expected that the last two motifs are most frequent, but the difference between them is surprisingly large and this is reproduced only by the EDR model; the CDR provides much closer probabilities for the two.

We also counted the number of fully connected subgraphs (cliques) with a certain size. This being a computationally costly procedure, we covered only the clique sizes from 3 to 8, and we also searched for the largest ones above 40 going up to 43. In the neuropil network, the largest cliques have 43 nodes; there are 31 of these including in total 53 of the brain areas (Figure 5I). Together, these form an extremely dense core (density 98%), being similar to the core–periphery structure noticed in the macaque (Ercsey-Ravasz et al., 2013; Markov et al., 2013). Comparing this to the random model networks generated with*λ* = 0 mm^−1^ (CDR) and 33 mm^−1^ (EDR), we see that the CDR model drastically underestimates the number of cliques (for large cliques, the values are all 0), while the EDR model gives good estimation in case of small cliques (Figures 5G and 5H). Nevertheless, for the largest ones, even the EDR model cannot reproduce the huge number of cliques. This shows these are specific structures that even if their presence is supported by this geometrical rule, there must be other reasons for which these are so frequent.

### Comparing Weighted Network Properties

**Figure 6.**
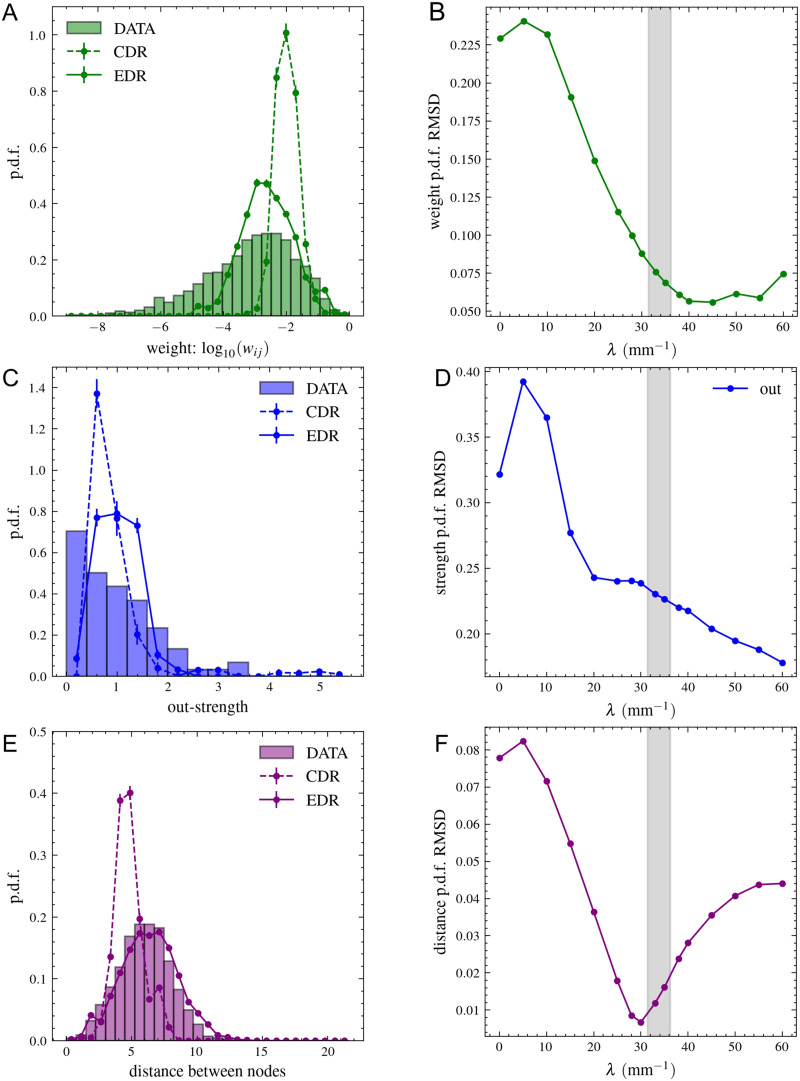
Comparing weighted network properties of the neuropil network and EDR model. (A) The distribution of logarithmic weight values (log_10_*w*_*ij*_) is slightly wider in the dataset, but clearly the CDR gives a much worse estimation than the EDR model with *λ* = 33 mm^−1^. (B) The RMSD calculated for the link length distributions between data and model is shown as a function of *λ*. (C) Distribution of out-strength (weighted out-degree; see the Materials and Methods section) of nodes. In-strength is not shown, because it is 1 for each node, the weighted matrix being normalized. (D) RMSD for out-strength distributions as a function of *λ*. (E) Distances between all node pairs are calculated in the network using the *l*_*ij*_ = −log*w*_*ij*_ length values. Their distribution agrees well with the *λ* = 33 mm^−1^ EDR model. (F) The RMSD calculated between the data and model for the node distance distribution as a function of *λ*.

As one can see, most binary network properties are reproduced surprisingly well by this one-parameter EDR network model. The next step is to consider weighted properties. Here, we must take into consideration that connection weights between neurons (and therefore weights between neuropils derived from these values) are not as precise, as they are based on predictions provided by convolutional neural network models. Additionally, the five-synapse threshold considered may eliminate many true (even if weak) connections, making link weights between neuropils slightly weaker. Indeed, looking at the link weight distribution (specifically the distribution of log_10_*w*_*ij*_ values in Figure 6A), we can see that the distribution in the data has a longer tail, including weaker connections (smaller log_10_*w*_*ij*_ values) than the one reproduced by the*λ* = 33 mm^−1^ EDR model. Nevertheless, the two distributions are not far apart; their characteristic shape is a normal distribution (*w*_*ij*_ being lognormal), and as shown in Figure 6B, the optimal *λ* predicted would be slightly larger, around 40 mm^−1^.

Figure 6C and Figure 6D provide a similar illustration for the out-strength (weighted out-degree) distribution of areas. Again, an unnaturally large peak of small strength values is observed in the data. The EDR model reproduces the mean value of the distribution fairly well but provides a shape closer to a normal distribution. However, as shown in Figure 6E and Figure 6F, the distribution of distances between nodes (the shortest path length calculated using the −log*w*_*ij*_ length values; see the Materials and Methods section) is reproduced surprisingly well by the EDR model. We argue that this is because strong connections are well predicted by the algorithms and are more precise. Errors typically have more drastic effects on weaker connections, which do not become part of the shortest paths in the network.

**Figure 7.**
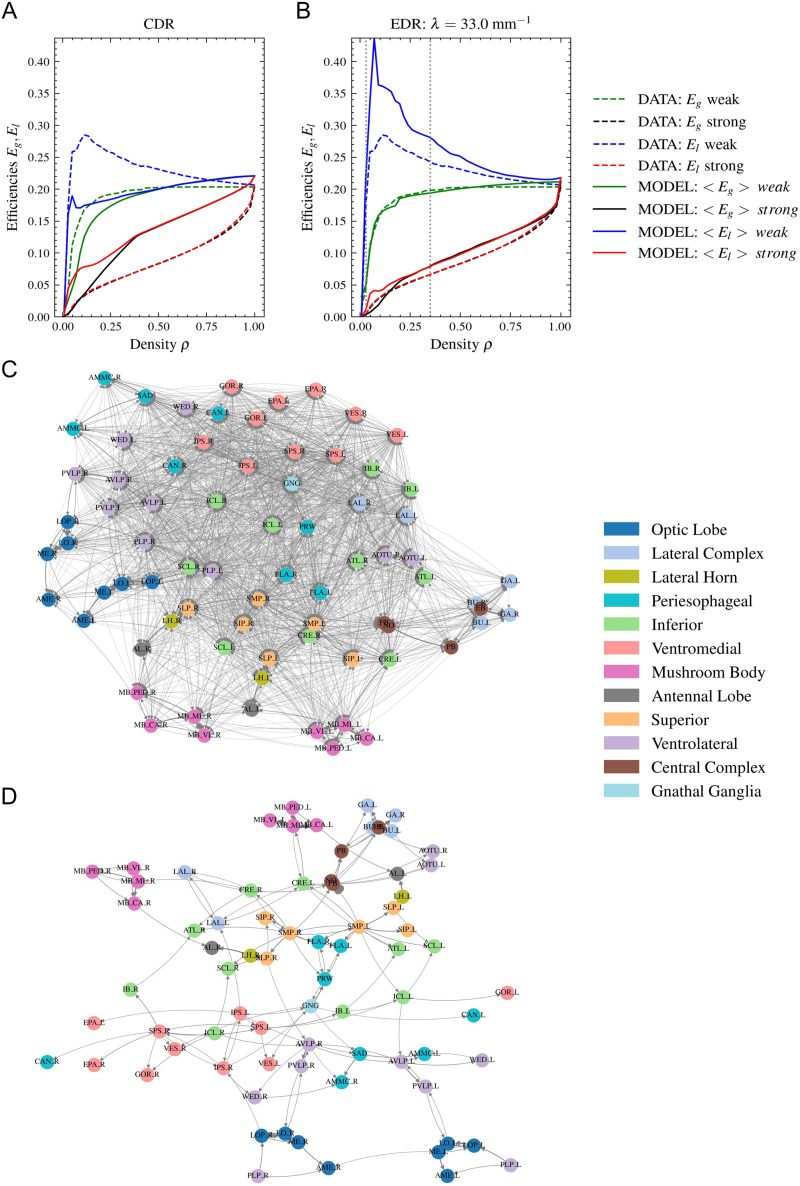
Communication efficiency and backbone of the projectome. The global and local communication efficiency values are calculated in the network as a function of density when taking out the links one by one ordered by their strength. Two cases are considered, taking out the weakest or the strongest links first (see legends). The results obtained from the data are represented with a dashed line; the averages obtained from the 1,000 model networks in the (A) CDR and (B) EDR with *λ* = 33 mm^−1^ are shown with a continuous line. (C) The strongly connected and (D) weakly connected backbone (with densities of 35% and 3%, respectively, shown on B with dotted lines) of the projectome is plotted using the Kamada–Kawai force-layout algorithm.

There are two other network measures based on the shortest path lengths between nodes that characterize the communication efficiency in a network. These are called global and local communication efficiency (Latora & Marchiori, 2003; Vragović et al., 2005; definitions in the Materials and Methods section). Similar to the analysis performed by Ercsey-Ravasz et al. (2013) for macaques and Horvát et al. (2016) for mice, we plot these efficiency values as a function of network density, while removing links ordered by their weight. In the *Drosophila* neuropil network, we observe the same behavior as in macaques and mice: When strong connections are removed first, both efficiency values decrease rapidly; when weak connections are removed first, the global efficiency remains almost constant until a low density is reached and the network falls apart, while the local efficiency even increases, showing a large peak at small densities. Similar to macaques and mice, even if the curve is not reproduced precisely, this type of behavior is observed only when applying the EDR model. The CDR (*λ* = 0 mm^−1^) does not show this phenomenon (Figures 7A and 7B). As explained in case of macaques and mice, this behavior is supported by the hierarchical modular structure of the network produced by the EDR.

In Figure 7C and Figure 7D, we plot the strongly connected backbone of the network (where there are paths in both directions between any pair of nodes) with a density of 35% and the weakly connected backbone of the network (where there are paths between any pair of nodes, but the directions of links are neglected) with a density of 3%. These two densities are indicated with a dotted line in Figure 7B. One can notice that the peak of the local communication efficiency is exactly before reaching the weakly connected backbone. The figures were produced using the NetworkX software and the Kamada–Kawai algorithm, which is a spring-based, force-directed algorithm (Kamada & Kawai, 1989). The nodes have been colored based on their classification into lobes in the FlyWire database. We can see how nodes with the same color usually group together, indicating that structural clusters have functional roles in the brain.

### Some Functionally Relevant Properties Cannot Be Reproduced by the Geometric Model

Naturally, we cannot expect every property of the projectome to be replicated by a random network model. The EDR model takes into account only the distance matrix between areas, the total number of connections, and the distance rule itself; everything else is random. As we have shown, it reproduces many *general* topological properties, but we cannot expect it to reproduce the specific properties of areas. For example, if we compare the degree not by looking at the general form of the degree spectrum (by sorting the areas in descending order separately for the data and model as in Figure 4), but specifically comparing the degrees of areas (for the dataset and model average and standard error over the 1,000 random networks with *λ* = 33 mm^−1^) as seen in Supporting Information Figure S7A, the model does not reproduce the degree of each node.

We also looked at the modular structure of the generated EDR model networks. Because each random network will have a slightly different modular structure (the example shown for one random network in Supporting Information Figure S7B), we first searched for these optimal structures separately for each network (fixing the number of clusters to four). Next, we have built a so-called contingency matrix from these optimal structures showing what is the probability of two nodes being in the same cluster for the ensemble of 1,000 random networks. This matrix is then again clustered with the hierarchical clustering method applied on the dataset (Supporting Information Figure S7C). Similarly to the dataset, we can notice that brain areas are grouped locally together in space forming a hierarchically modular structure (see Figure 3 and Supporting Information Figure S7C). This is generally expected from the EDR model due to the geometrical rule included in it. However, we cannot expect the model to reproduce precisely the modules detected in the neuropil network of the experimental data.

**Figure 8.**
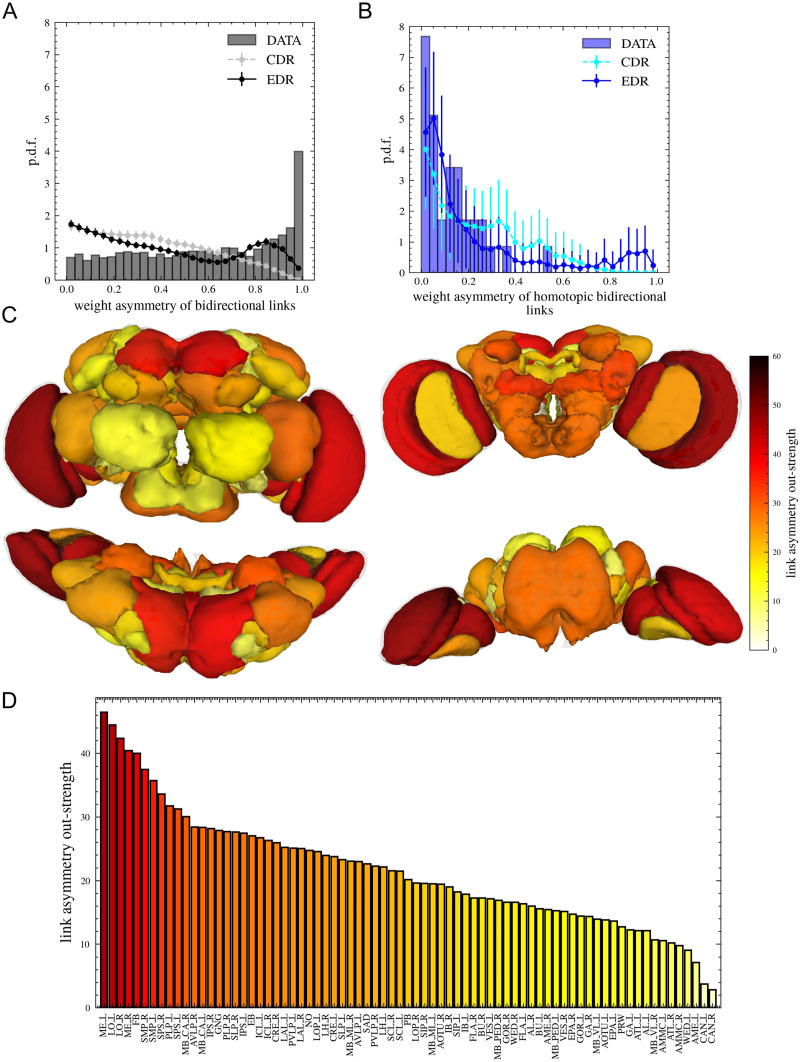
Asymmetry of connection weights. (A) Probability distribution of asymmetries for all node pairs connected with bidirectional links. (Error bars are not visible because SD values are small.) (B) Probability distribution of asymmetries for homotopic links connecting the same functional regions between left and right hemispheres. (C) Brain regions are colored based on the asymmetry out-strength calculated on the data set (for the definition, see the Materials and Methods section). Front, back, top, and bottom views are shown. Areas with high/low asymmetry out-strength are expected to be at the bottom/top of the hierarchy. A similar figure for the asymmetry in-strength is shown in Supporting Information Figure S9. (D) Ranking of neuropils based on their asymmetry out-strength is shown on a bar plot.

The importance of the EDR being applied as a null model lies in its ability to facilitate the detection of interesting, functionally relevant properties that are not a direct consequence of physical structure and geometry. One such, more general property of the projectome is the asymmetry of link weights between node pairs. For nodes *i* and *j*, we define this measure as the relative difference of weights in the two directions: $ASYM_{ij}=\frac{|w_{ij}-w_{ji}|}{w_{ij}+w_{ji}}\;$(see the Materials and Methods section). We calculate this measure for all node pairs, but obviously for pairs connected with a link only in one direction, this value is 1. The number of these unidirectional links has been well predicted by the EDR model (Figure 5A). In Figure 8A, we show the histogram of the asymmetries only for the bidirectional links. As we can see, there is a large peak at high values (close to 1), and there are few symmetric links with values close to zero. Neither the CDR with *λ* = 0 mm^−1^ nor the EDR with *λ* = 33 mm^−1^ reproduces the characteristic shape seen in the data. These geometric models, being based on the symmetric Euclidean distance matrix between neuropils, do not favor the formation of strongly asymmetric link pairs. The model predicts much lower probabilities for high asymmetry values. Nevertheless, these asymmetries are present in the brain and are probably important in determining the functional hierarchy of brain areas (Felleman & Van Essen, 1991; Hilgetag et al., 1996; Hochstein & Ahissar, 2002).

Figure 8A is a histogram including all bidirectional links, but we were curious if anything changes when looking at area pairs from the same hemisphere (ipsilateral links; Supporting Information Figure S8A) or area pairs from different hemispheres (contralateral connections; Supporting Information Figure S8B). These show similar behavior; however, surprisingly, when looking at homotopic connections—link pairs connecting the left and right parts of the same functional areas—the distribution is completely different. These homotopic connections are much more symmetric and are relatively well predicted by the EDR model (Figure 8B). This supports the idea that this measure can be connected to the functional hierarchy of areas, the left–right parts of the same area being on the same hierarchical level; these are expected to be more symmetric.

In order to illustrate this functional hierarchy, we build a new network characterizing information flow with one single link between each pair of nodes. If *w*_*ij*_ > *w*_*ji*_, then the link is directed from *i* to *j*, otherwise from *j* to *i*, and we use the asymmetry values as link weights of this new network. Calculating the in- and out-strength (weighted degree) of areas in this network, we characterize how strong is the outgoing/incoming asymmetry. In Figure 8C, we color the areas according to their out-strength of asymmetries, showing the map from front, back, top, and bottom. There are several observations supporting that this measure gives information about hierarchy: (a) The ranking of areas is strongly symmetrical. (b) We can observe how the areas from the optic lobe (ME, LO) have the largest out-strength being at the lowest level of hierarchy, where information mainly comes in from sensory input and is forwarded to higher level areas for processing (as shown in Figure 8D). The areas at the top of the hierarchy with the lowest out-strength seem to be CAN, AME, WED, AMMC, MB_VL, and so forth. Usually, areas that are at the bottom of the top list for the asymmetry out-strength are at the top of the list for the asymmetry in-strength (cf. Figure 8D with Supporting Information Figure S9B); this also supports that this list could give information about the list of hierarchy between areas. The same brain map based on the in-strength of asymmetries is shown in Supporting Information Figure S9A.

Asymmetries are expected to be formed during brain development, and a potential cause is that the brain itself is not a closed system; input/output signals are arriving/exiting each area in different amount. This suggests that another possible way to investigate if these asymmetries are connected to functional hierarchy would be to count the number of presynapses of incoming (afferent) neurons in each area. We expect that areas at the bottom of the hierarchy should have a large number of incoming synapses from outside the brain. In Supporting Information Figure S10A, we colored the brain areas based on these afferent presynapse counts, the ordering shown in Supporting Information Figure S10B. We can notice some similarities with Figure 8C especially from the back and top look of the brain. Even if these similarities are not strong, these large differences (six orders of magnitude) between the incoming information are certainly expected to build up a direction of information flow that during development can cause asymmetries between the weights of bidirectionally connected areas. Supporting Information Figure S11A shows the counts based on the postsynapses of efferent neurons, trying to estimate the information amount exiting the brain areas (the ranking of areas is shown in Supporting Information Figure S11B).

## DISCUSSION

The FlyWire project provides a comprehensive open-access dataset with a neuron-level mapping and detailed information on the *Drosophila* brain. In this paper, we used these data to demonstrate that the EDR-based, region-level network model, known to apply in macaques, mice, and rats, also holds true for the *Drosophila*. By analyzing the available neuron tree structures, we measured the decay rate of the EDR using both real cable lengths between the axonal and dendritic arbors, and Euclidean distances. We estimated the decay rate to be in the interval *λ_d_min__* = [18.4, 21.5] mm^−1^ for real cable lengths and *λ_ED_min__* = [31.4, 36.2] mm^−1^ for Euclidean cable lengths. This factor is crucial for understanding the *Drosophila*, whereas in larger brains (such as those of macaques and mice), the difference between these measurements is negligible.

Next, we studied the network of neuropils (brain regions) and applied the EDR-based network model, similar to previous studies on macaques and mice (Ercsey-Ravasz et al., 2013; Horvát et al., 2016). We found that the EDR model accurately predicts most binary properties, such as degree distributions, uni- and bidirectional links, clustering coefficient, average path length, and triangular motifs. However, the model underestimates the huge number of large cliques (completely connected subgraphs). In the neuropil network, the largest clique consists of 43 out of 75 nodes, and there are 31 such cliques, involving 53 nodes in total. These cliques are crucial for the brain’s modular structure and hierarchy. Like in macaques and mice, these form a dense core of the network. While the geometric rule supports this property, as the CDR model cannot reproduce even small cliques, the EDR model with *λ* = 33 mm^−1^ reproduces small cliques but still underestimates the largest ones.

For weighted properties, we delved deeper than previous studies, comparing weight distributions, out-strength and node distance distributions, as well as local and global communication efficiencies. The qualitative behavior is well reproduced, and the behavior of communication efficiency as a function of density is similar to that found in macaques and mice. However, there are several quantitative differences in the link weight and out-strength distributions. We suggest this may be due to imprecisions in connection weights between neurons determined by convolutional neural networks and the applied five-neuron threshold (Buhmann et al., 2021), which can eliminate some true links, weakening connections between neuropils. This argument is supported by the fact that the node distance distribution is accurately reproduced by the model, as shortest paths typically involve strong links that are less affected by errors.

An interesting property of the neuropil network, not directly studied before and not reproduced by the geometrical model, is the asymmetry of weights. By examining all bidirectional links, we calculated the relative difference between the weights in the two directions. While a geometrical model typically does not produce strong asymmetries (being based on the symmetric Euclidean distance matrix between neuropils), these are surprisingly prevalent in the brain and likely important for its functional hierarchy. Another surprising finding is that homotopic connections—links between the left and right sides of the same functional areas—are much more symmetric and align well with the model. This supports the idea that strong asymmetries relate to functional hierarchies, as the left and right sides of the same area are expected to be on the same level of the hierarchy. These asymmetries could be used to develop functional hierarchical models, as attempted before for the visual processing system (Felleman & Van Essen, 1991; Hilgetag et al., 1996; Hochstein & Ahissar, 2002). Here, we made a first attempt to build a hierarchy by using the total asymmetry out- and in-strength values to highlight the ranking of areas. We also tried to relate this hierarchy to the number of synapses of afferent/efferent neurons entering/exiting the areas, based on the hypothesis that areas at the bottom of functional hierarchy have a larger amount of incoming information. This deserves more detailed future studies, but we believe this shows that the comparison of real region-level structural brain networks with the EDR-based null model can reveal information about the network’s functionally relevant properties. This ability to dissect the nuances of brain structure and function not only enhances our understanding of neural architecture but also opens avenues for future research aimed at exploring the evolutionary implications of these organizational principles across different species.

Previous empirical studies demonstrate that the EDR is valid across various species. Theoretical results also support the presence of EDR in densely packed physical networks, such as the brain (Pósfai et al., 2024). While the EDR itself is a neuron-level information, the EDR-based network model reproduces surprisingly well many properties of region-level networks in very different species, including insects, rodents, and primates. Therefore, we argue that the EDR-based model is an appropriate null model for analyzing structural brain networks on meso- and macroscales (level of brain regions). This model effectively predicts many topological properties, suggesting that most are consequences of geometry and physical structure. Comparing structural networks to the EDR-based null model helps identify functionally relevant features that are not just due to geometry. For example, we highlighted the asymmetry of connection weights, which are likely crucial for forming the hierarchy of functional brain regions. To facilitate future studies, we have made the codes generating EDR model networks available on GitHub (https://github.com/bpentek/EDRmodel).

## MATERIALS AND METHODS

### The *Drosophila* Database

In recent years, the field of connectomics has seen advancements due to improvements in neuroimaging technologies and developments in machine learning/artificial intelligence/neural networks. In 2018, Zheng et al. successfully mapped the connectome of *Drosophila melanogaster* (commonly known as the fruit fly), a model organism in biology since the early 20th century. They developed a specialized ssTEM that captured images of the adult female *Drosophila* brain with a resolution of just a few nanometers. Their research produced a dataset of approximately 7,000 images, totaling about 106 terabytes, which has been made publicly available (Zheng et al., 2018).

Buhmann et al. utilized this database to predict the chemical synapses in the adult *Drosophila* brain using a large convolutional neural network. The authors found that their model performed best for one-to-many synapses, where a single presynaptic site connects to multiple postsynaptic sites. Considering the polyadic nature of insect synapses (where multiple synapses can connect the same pair of neurons), they determined that setting a threshold of at least five synapses/connection allowed for highly accurate predictions of the remaining neural connections (Buhmann et al., 2021). This thresholding can be reasoned also from a more biological perspective, as the stronger connections (with > 4 synapses) can be considered physiologically more significant, also present across individuals (Dorkenwald et al., 2024).

The FlyWire project established by Dorkenwald et al. (2022) enabled citizen scientists from all around the world to contribute to the reconstruction of the *Drosophila* connectome, by proofreading automatically traced neurons. Combined with the predictions made by Buhmann et al. and Heinrich et al., this project resulted in the largest fully mapped connectome to date with 139 thousand proofread neurons, 2.7 million thresholded connections with a total of 34 million synapses. Among other information, the FlyWire database also includes the atlas of neuropils (brain regions) developed by Ito et al. (2014). The complete dataset is available to download through the *Codex* web-app (Matsliah et al., 2023; codex.flywire.ai) and *fafbseg-py* Python package (Dorkenwald et al., 2024; Schlegel et al., 2024; github.com/navis-org/fafbseg-py).

For our study, we utilized the connectivity matrix and the neuron classification table published on the Codex web interface to construct the *neuropil projectome* from the connections between the intrinsic neurons. The neuron skeletons (spatial graph structures) with detected somas were also downloaded from *Codex*; the pre- and postsynaptic sites were attached to it using the *fafbseg* Python package. Additionally, the centroids of the neuropil meshes were downloaded from the *fafbseg* package, which are based on data originally published by Jenett et al. (2012). This information was needed for constructing the EDR model of the neuropil projectome. We used the latest data release available at the time when beginning our study, snapshot 783 from October 2023.

### Neuropil Projectome Construction

We constructed the connectome at the level of neuropils (also referred to as the projectome) using the algorithm developed by the FlyWire project team (Dorkenwald et al., 2024; Lin et al., 2024). This method is based on two assumptions about the information flow between neuropils:

1. The information flow through a single neuron can be expressed probabilistically by taking the fraction of its total synapses present in a given neuropil.
2. Incoming and outgoing information flows through the neuron are considered independent events.

For *N* neuropils and a single neuron, two separate vectors can be constructed to represent the fractions of incoming and outgoing synapses in the different regions, respectively. The tensorial product of these two vectors yields an *NxN* matrix, where each element *W*_*ij*_ represents the probability of the neuron having incoming information in neuropil *i* and outgoing in neuropil *j*. Summing these matrices for each neuron produces the connectivity matrix of the neuropil projectome, with higher matrix values indicating stronger connections between two neuropils.

Given the variability in neuropil sizes and the fact that larger neuropils typically contain more synapses, normalizing the weight matrix appears to be an effective approach for accurately representing overall structural connectivity. In line with previous studies (Ercsey-Ravasz et al., 2013; Horvát et al., 2016), we opted to normalize the weight matrix by its columns. Thus, the normalized element *w*_*ij*_ of the weight matrix reflects the probability of information flow between neuropils *i* and *j*.

### Hierarchical Clustering Method

The *scipy* library offers a vast number of options to perform hierarchical clustering in a bottom-up approach (agglomerating clusters in each step). One example of this family of agglomerative algorithms is the Ward’s method, which aims to minimize the variance within the clusters and uses the Euclidean distance metric (Murtagh & Legendre, 2014; Ward, 1963). Other popular agglomerative methods for determining distances between the newly formed clusters include the complete method (Farthest Point Algorithm), the average method (UPGMA Algorithm), and many more (Murtagh & Contreras, 2012). A wide variety of options is available also for the distance metric; in a network science context, notable examples include the cosine and the correlation similarity metrics (Murtagh & Contreras, 2012; Newman, 2010).

In our specific case, we have the 75 × 75 weighted adjacency matrix representing the link lengths (*l*_*ij*_ = −log*w*_*ij*_ values) between the nodes in the *Drosophila* projectome. The data points we chose to cluster are given by 75-dimensional vectors represented by the columns of the matrix. Therefore, in simple terms, the clustering groups together neuropils with similar vectors of incoming information flow.

### Network Measures

–Degree of a node in a network gives essentially the number of its neighbors (edges connected to it; Newman, 2010). Mathematically, it can be expressed using the adjacency matrix *A*_*ij*_, which takes the value of 1 if there is a link going from *i* to *j*; otherwise, it is 0.

$$k_{i}=\sum_{j=1}^{N}A_{ij}$$

In a directed network, this value can be separated based on the direction of links (incoming or outgoing); therefore, we can talk about the in-degree and out-degree of a node (Newman, 2010).

Similarly, in a weighted network, the so-called in- and out-strengths (weighted degrees) can be defined, by using the weighted adjacency matrix in the sum.

–  Average (binary/unweighted) path length measures the typical number of links in the shortest paths connecting two nodes in the network (Newman, 2010). In small-world networks, this average path length is relatively short compared with the total number of nodes, indicating that nodes are generally accessible from one another with just a few steps (Watts & Strogatz, 1998).
  $$APL=\frac{1}{N\left(N-1\right)}\sum_{i\neq j=1}^{N}d_{ij}$$
–  Clustering coefficient of a node can be thought of as the probability that two neighbors of the node are connected (Newman, 2010). Overall in the network, we calculate the average for all nodes.
  $$CC=\frac{1}{N}\sum_{i=1}^{N}CC_{i}=\frac{1}{N}\sum_{i=1}^{N}\frac{1}{k_{i}\left(k_{i}-1\right)}\sum_{j,k\in \left\{i\right\}}A_{jk}$$

Here, *{i}* denotes the neighborhood of node *i* (subgraph consisting of its neighbors).

–  Triangular motifs in a network are subgraphs consisting of three nodes. In the directed case, there are a total of 16 possible edge configurations between the three nodes. These triangles have been found to be characteristic building blocks of different real-world networks (Milo et al., 2002).
–  A *k-clique* is a fully connected subgraph consisting of *k* nodes. In the case of directed networks, fully connected means that there are edges/links between all node pairs in both directions (Newman, 2010).
–  The length of a link is defined as: *l*_*ij*_ = −log*w*_*ij*_. This approach was already used both in structural (Ercsey-Ravasz et al., 2013; Markov et al., 2013) and functional brain networks (Varga et al., 2024; Wandres et al., 2021). The argument is that the link weight is proportional to the probability of information transfer, so the probability for information to pass on from node *i* to node *j* through node *k* would be: *w*_*ij*_ *w*_*jk*_. Using the logarithmic form, these become additive: −log*w*_*ik*_ = −log*w*_*ij*_ − log*w*_*jk*_ = *l*_*ij*_ +*l*_*jk*_, and we can calculate the shortest paths (weighted distances) in the network that will indicate the most probable paths for information transfer (Ercsey-Ravasz et al., 2013; Markov et al., 2013).
–  Distance (or resistance, *r*_*ij*_) between two nodes in the network is the length of the shortest path. Its inverse is sometimes called “conductance” (in analogy with physical circuits; Ercsey-Ravasz et al., 2013; Markov et al., 2013).
–  Global communication efficiency is defined as the average of the inverse resistance (“conductance”), here defined as the length of the shortest path between all node pairs (Latora & Marchiori, 2003):
  $$E_{g}=\frac{1}{N\left(N-1\right)}\sum_{i\neq j=1\;}^{N}\frac{1}{r_{ij}}$$
–  Local communication efficiency is (Vragović et al., 2005):
  $$E_{l}=\frac{1}{N}\;\sum_{i=1}^{N}{E_{l}}_{i}=\frac{1}{N}\;\sum_{i=1}^{N}\frac{1}{k_{i}\left(k_{i}-1\right)}\sum_{j,k\in \left\{i\right\}}\frac{1}{r_{jk/i}}$$

where *{i}* indicates the set of neighbors of node *i*; *j* and *k* are neighbors of *i*; *r*_*jk*/*i*_ is the shortest path between *j* and *k* after the removal of node *i* and its links from the graph; and *k*_*i*_ is the degree (number of neighbors, links) of node *i*. This gives information on how efficient is the communication between the neighbors of node *i*, but without using the links connecting them to node *i*. This local measure is averaged over all nodes (the first sum in the formula).

–  The backbone of a weighted network is obtained by sequentially removing its weakest links while maintaining network connectivity (Ercsey-Ravasz et al., 2013). This means that there remains at least one path between every pair of nodes. For directed networks, this connectivity can be defined in two ways: a *strongly connected backbone*, where paths must exist in both directions between all pairs of nodes, or a *weakly connected backbone*, where the direction of edges is ignored.
–  The Kamada–Kawai layout is a method for visualizing networks in space using a force-directed algorithm. In this approach, the network is modeled as a system where each pair of nodes is connected by a virtual spring with a strength inversely proportional to the square of the graph-theoretic distance between them (Kamada & Kawai, 1989). This algorithm seeks to minimize the total energy of the system by adjusting the positions of the nodes so that the Euclidean distances between them closely match the desired graph distances. As a result, nodes that are close within the network (i.e., connected by short paths) are placed near each other in the visualization, while nodes that are distant are positioned further apart.
–  The weight asymmetry of a link between two nodes *i* and *j* is defined as the relative difference between the weights of the links in both directions:
  $$\;ASYM_{ij}=\frac{|w_{ij}-w_{ji}|}{w_{ij}+w_{ji}}$$

This metric quantifies the degree of asymmetry in the weights of bidirectional links between nodes. It ranges from 0 to 1, a lower value indicates stronger symmetry, and a higher one greater asymmetry. It can be also defined for unidirectional links, in which case this measure is equal to 1.

–  Based on the previous asymmetry metric, a new network can be constructed describing asymmetries with unidirectional links only: If *w*_*ij*_ > *w*_*ji*_, we insert a directed link pointing from *i* to *j* with a weight of *ASYM*_*ij*_; in the other case, the direction of the link is the opposite (from *j* to *i*). This approach ensures that the direction of the link corresponds to the predominant direction of connection between the nodes. Then, the overall strength of asymmetries for a node can be measured by computing the in- and out-strengths (weighted degrees) in this newly formed network. Nodes that have mainly outgoing links (large out-strength of asymmetries) are at the bottom of the functional hierarchy (e.g., optic lobe): They obtain information from sensory inputs outside the brain and forward it to more higher level (central) areas for processing; those have mainly incoming connections.
–  The cosine similarity between two nodes uses the rows (or columns) of the adjacency matrix to represent two vectors, with the cosine of the angle between them being the similarity measure (Newman, 2010).
  $$\sigma_{ij}=\frac{\sum_{k}A_{ik}A_{kj}}{\sqrt{\sum_{k}A_{ik}^{2}}\sqrt{\sum_{k}A_{jk}^{2}}}$$
–  The Pearson correlation similarity also treats the rows (or columns) of the adjacency matrix as vectors, for which the well-known formula of Pearson’s *r* can be written (Newman, 2010).
  $$r_{ij}=\frac{\text{cov}\left(A_{i},A_{j}\right)}{\sigma_{i}\sigma_{j}}=\frac{\sum_{k}\left(A_{ik}-\langle A_{i}\rangle \right)\left(A_{jk}-\langle A_{j}\rangle \right)}{\sqrt{\sum_{k}{\left(A_{ik}-\langle A_{i}\rangle \right)}^{2}}\sqrt{\sum_{k}{\left(A_{jk}-\langle A_{j}\rangle \right)}^{2}}}$$

### Other Statistical Measures

–  RMSD of two different samples is defined as:
  $$\text{RMSD}=\sqrt{\frac{1}{n}\sum_{i=1}^{n}{\left(x_{i}-y_{i}\right)}^{2}}$$

In this context, RMSD is used to measure the differences between samples (properties of the brain network) derived from the real dataset and those predicted by the model.

### Software Packages

The database of the *Drosophila* connectome was downloaded from the Codex web interface (Matsliah et al., 2023) and fafbseg python package (Dorkenwald et al., 2024; Schlegel et al., 2024). A large part of the network analysis was performed using igraph library (Csárdi et al., 2024); for visualization, the NetworkX package was used (Hagberg et al., 2008). Neuron skeletons were processed with the navis package (Philipp Schlegel et al., 2024); the neuropil plots were created in the neuroglancer environment (Maitin-Shepard et al., 2021). Hierarchical clustering was performed using the scipy implementation (Virtanen et al., 2020), and our own code for EDR model network generation is available on GitHub (https://github.com/bpentek/EDRmodel).

## Acknowledgments

This study was supported by the Romanian National Authority for Scientific Research and Innovation, CNCS-UEFISCDI, projects: PN-III-P4-PCE-2021-0408 (M. E-R., B.P.), ERANET-FLAG-ERA-ModelDXConsciousness (M.E-R), ERANET-NEURON-2-UnscrAMBLY (M.E-R), ERANET-NEURON-2-IBRAA (M.E-R), ERANET-NEURON-2-RESIST-D (M. E-R., B.P.), FLAGERA-JTC2023-MONAD (M. E-R, B.P), PN-IV-P2–2.1-TE-2023-1548 (M. E-R.). B.P. was also funded by a student research scholarship of the Babes-Bolyai University nr. 36265/24.11.2023, the Federation of Hungarian Universities from Cluj-Napoca and the Bethlen Gábor Association, and The Bolyai Society from Cluj-Napoca. This work was also supported by the Collegium Talentum Programme of Hungary (B.P., M.E-R.).

FlyWire dataset including the fully mapped connectome, reconstructed neurons, etc.; available on their website (codex.flywire.ai) and through their Python API (github.com/navis-org/fafbseg-py):

- JFRC2 dataset including the spatial information of neuropils (coordinates, etc.); available also through the *fafbseg-py* API (github.com/navis-org/fafbseg-py).
- EDR model generation code implemented in C, available on GitHub (github.com/bpentek/EDRModel).

## Supporting Information

Supporting information for this article is available at https://doi.org/10.1162/netn_a_00455.

## Author Contributions

Balázs Péntek: Data curation; Formal analysis; Investigation; Methodology; Software; Validation; Visualization; Writing – original draft; Writing – review & editing. Maria Ercsey-Ravasz: Conceptualization; Funding acquisition; Investigation; Methodology; Project administration; Supervision; Writing – original draft; Writing – review & editing.

## Funding Information

Maria Ercsey-Ravasz, CNCS-UEFISCDI, Award ID: PN-III-P4-PCE-2021-0408. Maria Ercsey-Ravasz, CNCS-UEFISCDI, Award ID: ERANET-FLAG-ERA-ModelDXConsciousness. Maria Ercsey-Ravasz, CNCS-UEFISCDI, Award ID: ERANET-NEURON-2- UnscrAMBLY. Maria Ercsey-Ravasz, CNCS-UEFISCDI, Award ID: ERANET-NEURON-2-IBRAA. Maria Ercsey-Ravasz, CNCS-UEFISCDI, Award ID: ERANET-NEURON-2-RESIST-D. Maria Ercsey-Ravasz, CNCS-UEFISCDI, Award ID: FLAGERA-JTC2023-MONAD. Balázs Péntek, Universitatea Babeş-Bolyai (https://dx.doi.org/10.13039/501100006347), Award ID: 36265/24.11.2023. Balázs Péntek, Collegium Talentum Program. Not Applicable, CNCS-UEFISCDI, Award ID: PN-IV-P2–2.1-TE-2023-1548.

## Supplementary Material



## TECHNICAL TERMS

Neuropils: Functional brain regions defined on the brain map of a *Drosophila*. These are the nodes in the region-level network.
Convolutional neural network: A machine learning algorithm, which is highly efficient especially in image analysis. In this case, it was applied to identify the location of pre- and postsynapses in the *Drosophila* brain.
Efferent neurons: Neurons inside the brain sending information outside the brain.
Projectome: The name of the region-level (neuropil) network in a *Drosophila*.
Strength: The weighted degree of a node, meaning the sum of weights of the links connected to the node. We can define separately out-strength and in-strength based on outgoing and incoming links.
Degree: The number of links of a node. In case of directed networks, we can separately count the outgoing links (out-degree) or incoming links (in-degree).
Clustering coefficient: In case of one node, this is the probability that two neighbors of the node are connected. Overall in the network, we calculate the average for all nodes.
Network density: The ratio between the existing links (*M*) and the maximum possible number of links, *N*(*N*–1) in case of a directed network with *N* nodes.
Afferent neurons: Neurons outside the brain sending information to the brain.
Shortest binary path length: The number of links in the shortest path connecting two nodes. The average shortest path length considers the average over all pairs of nodes in the network.
Afferent neurons: Neurons outside the brain sending information to the brain.
