# Supplementary material for: The exponential distance rule-based network model predicts topology and reveals functionally relevant properties of the Drosophila projectome
Source: Netw Neurosci. 2025 Jul 29;9(3):869–95. doi: 10.1162/netn_a_00455 (PMC12543305; doi:10.1162/netn_a_00455)
Supplement: Supplementary file 1 [file netn-9-3-869-s001.pdf]

Supporting Material

The exponential distance rule based network model predicts topology and reveals functionally relevant properties of the *Drosophila* projectome

Balázs Péntek, Mária Ercsey-Ravasz

Supplementary Figures

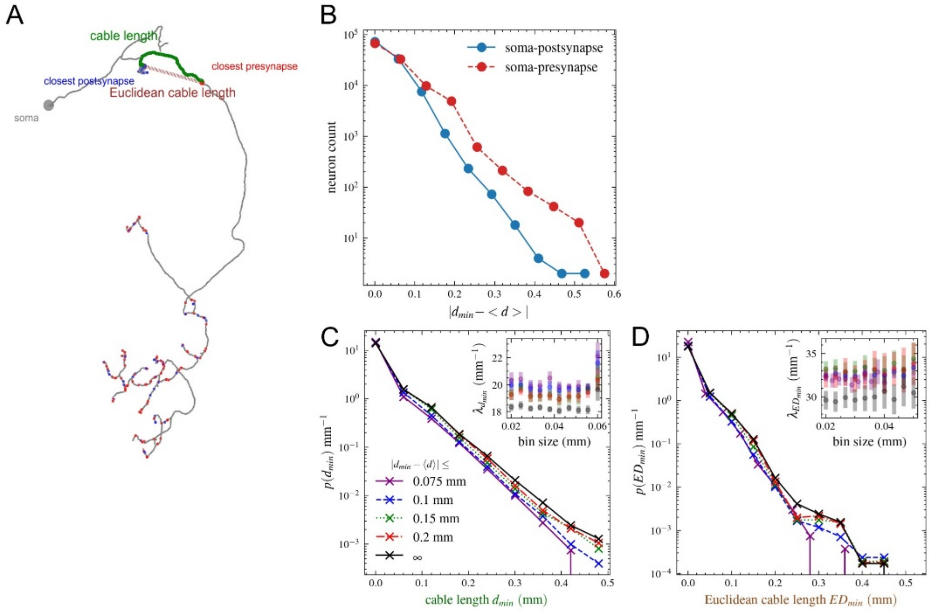

Supplementary Figure 1. Excluding errors with thresholding neurons & its effect on EDR. A) An example for non-traditional presynaptic points shown on the axon resulting in a much shorter measured cable length. B) Histograms of the absolute difference between minimum soma-pre-/postsynapse path length and the average. C) EDR of cable lengths for different upper threshold values of $|d_{min} - \langle d \rangle|$, applied both for pre- and postsynapses. Purple line shows the EDR for a threshold of 0.075 mm (57% of total neurons), fitting an exponential to the data results in an interval of $\lambda_{d_{min}} = [19.4, 23.1]\, mm^{-1}$. Blue line: 0.1 mm (72%), $\lambda_{d_{min}} = [19.1, 22.5]\, mm^{-1}$. Green line: 0.15 mm (89%), $\lambda_{d_{min}} = [18.4, 21.5]\, mm^{-1}$. Red line: 0.2

mm (96%), $\lambda_{d_{min}} = [18.5, 21.2]\ mm^{-1}$. Black line: no threshold (all 115,953 neurons), $\lambda_{d_{min}} = [17.8, 20.5]\ mm^{-1}$. D) Similarly to C), but for the EDR of Euclidean cable lengths. Purple line: 0.075 mm, $\lambda_{ED_{min}} = [30.5, 34.3]\ mm^{-1}$. Blue line: 0.1 mm, $\lambda_{ED_{min}} = [31.5, 34.4]\ mm^{-1}$. Green line: 0.15 mm, $\lambda_{ED_{min}} = [31.4, 36.2]\ mm^{-1}$. Red line: 0.2 mm, $\lambda_{ED_{min}} = [30.3, 36.3]\ mm^{-1}$. Black line: no threshold, $\lambda_{ED_{min}} = [28.0, 33.5]\ mm^{-1}$.

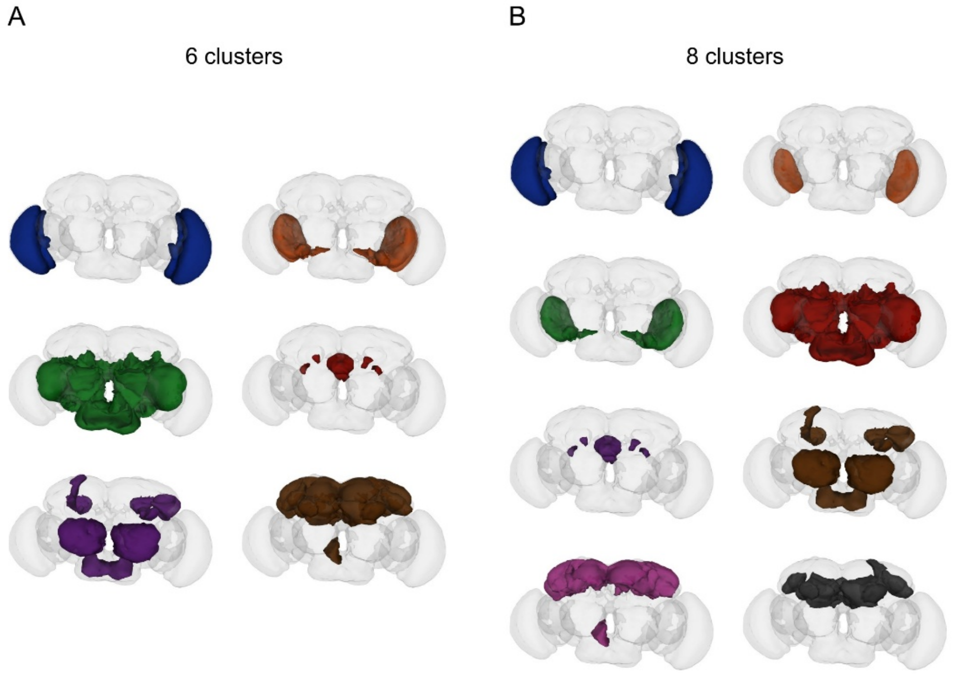

Supplementary Figure 2. Deeper look into the modular structure of the *Drosophila* network neuropil. A) The six largest clusters presented one by one from a front view. Going one level below on the dendrogram shown in Figure 3A, the cluster of the optical lobes (originally blue in Figure 3B,C) further divides into two groups, separating the medulla (blue) from the lobula & antennal mechanosensory motor center (orange). The division of the other cluster located at the top & frontal side of the brain (originally red on Figure 3B,C) introduces some asymmetry into the spatial structure, mainly by separating the left and right parts of the lateral horn, pedunculus and calyx parts of the mushroom body (purple and brown). B) Cutting the dendrogram even more below to get the 8 largest clusters, it can be observed that the lobular

plate (orange) gets separated from its original group. Also, the top side cluster gets further divided into two slightly asymmetric groups (pink and grey).

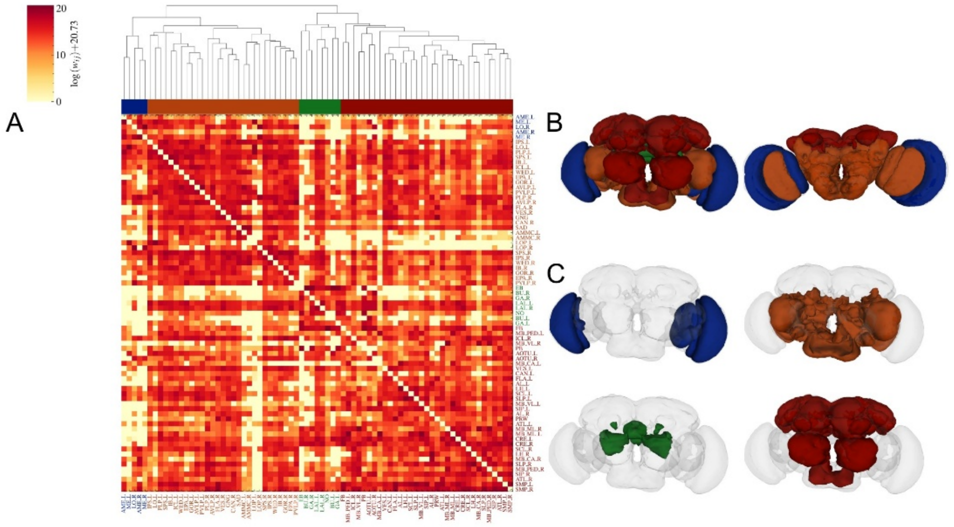

Supplementary Figure 3. Modular structure of the projectome for other hierarchical clustering methods. A) Weighted connectivity matrix with the order of areas & dendrogram provided by the average cluster linkage paired with correlation node similarity. B) Resulting four largest clusters from a front and back view. C) The four clusters visualized separately. A similar image is obtained as using Ward's method for clustering (Fig.3), main differences being the Antennal Mechanosensory and Motor Center and the Lobular Plate as well being separated from the optic cluster (blue); the Lateral Complex (with LAL included) fully clustered together with parts of the Central Complex (EB, NO). The Lobula joins the Cantle and Flange in being the only neuropils with their left and right parts placed into different clusters.

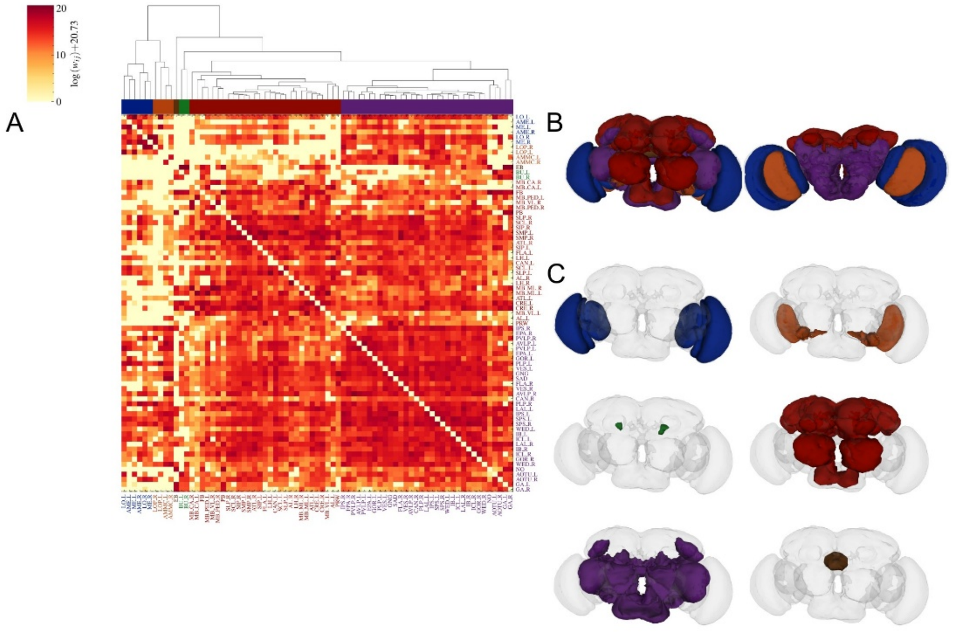

Supplementary Figure 4. Modular structure of the projectome for other hierarchical clustering methods. A) Weighted connectivity matrix with the order of areas & dendrogram provided by the complete cluster linkage paired with cosine node similarity. B) Resulting six largest clusters from a front and back view. C) The six clusters visualized separately. In accordance to the results obtained with Ward's method (Fig.3), the spatial structure of the clusters is localized and highly symmetric, aside from the parts of Cantle and Flange.

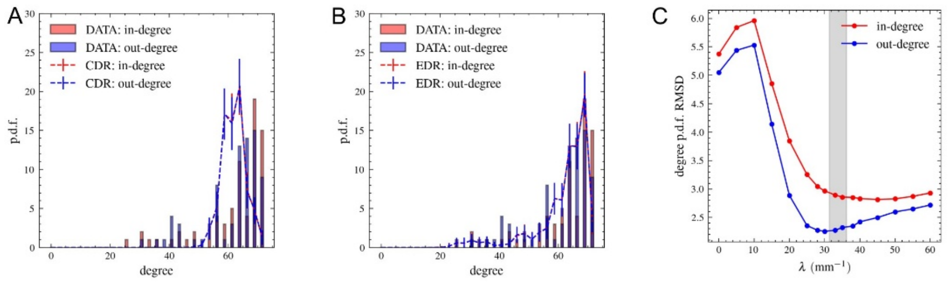

Supplementary Figure 5. Degree distributions: dataset vs. model. Having a small network with 75 nodes, these are relatively noisy. A) in- and out-degree (red and blue) of dataset (bar plots) and average values for CDR models with $\lambda = 0\ mm^{-1}$(dashed line; errorbar indicating standard deviation); B) similar to A), but for EDR model with $\lambda = 33\ mm^{-1}$; C) RMSD as a

function of λ, the gray interval showing the fitted values in Fig. 1C: $\lambda_{ED_{min}} = [31.4, 36.2]\ mm^{-1}$.

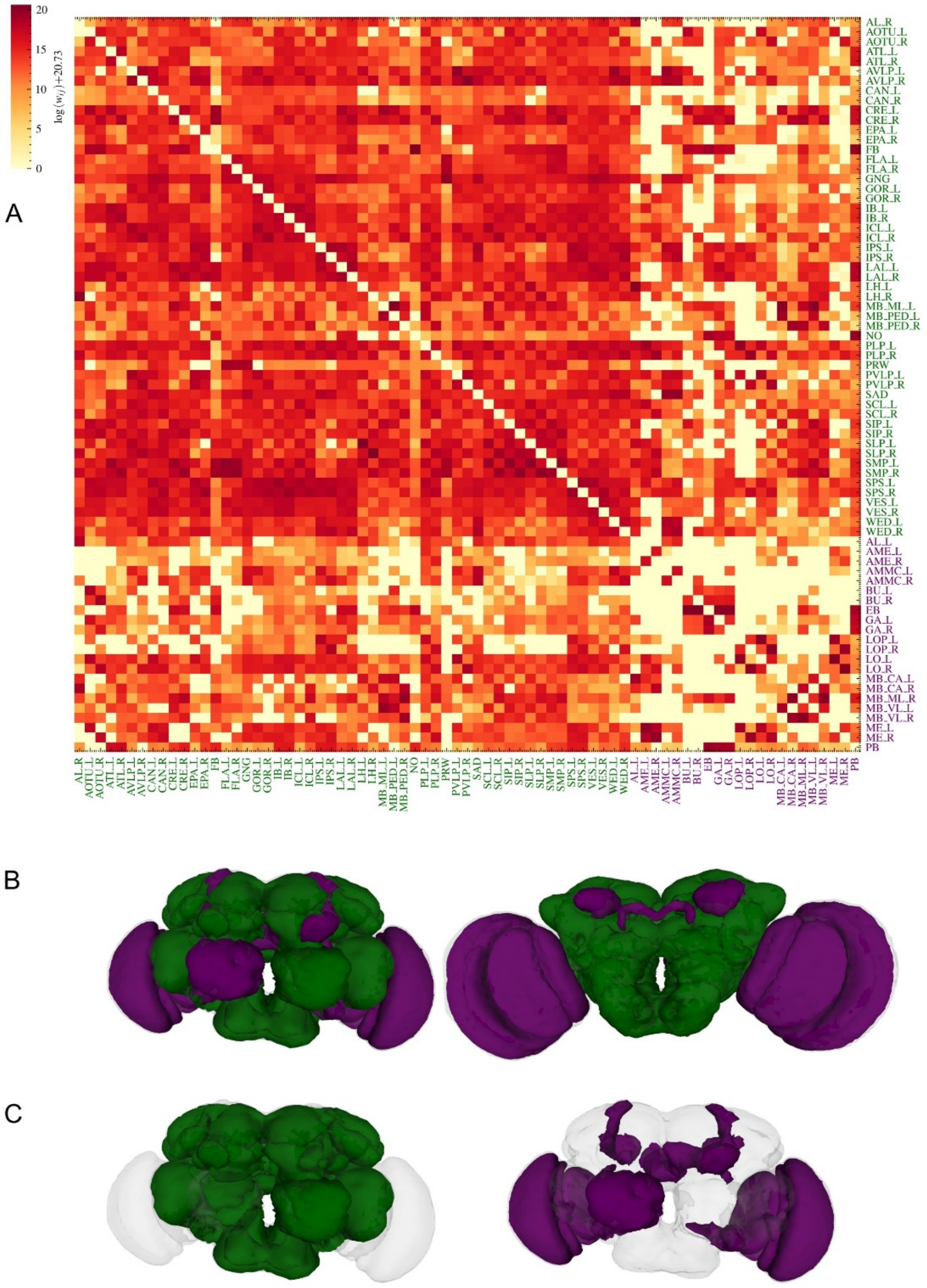

Supplementary Figure 6. Core-periphery structure based on the largest cliques in the *Drosophila* projectome. A) Matrix plot representing connection strength between neuropils. The rows and columns are ordered such as the nodes corresponding to the network core are

shown first (green labels), then the periphery (purple labels). The network core consists of 53 nodes in total, resulting from the set of nodes in the 31 largest cliques of size 43. We can see the large density and strong connections inside the core. B) The core (green) and peripheral (purple) neuropils shown on the *Drosophila* brain from a front and back view. C) The same two sets of neuropils shown separately on the *Drosophila* brain from a front view (core on the left, periphery on the right).

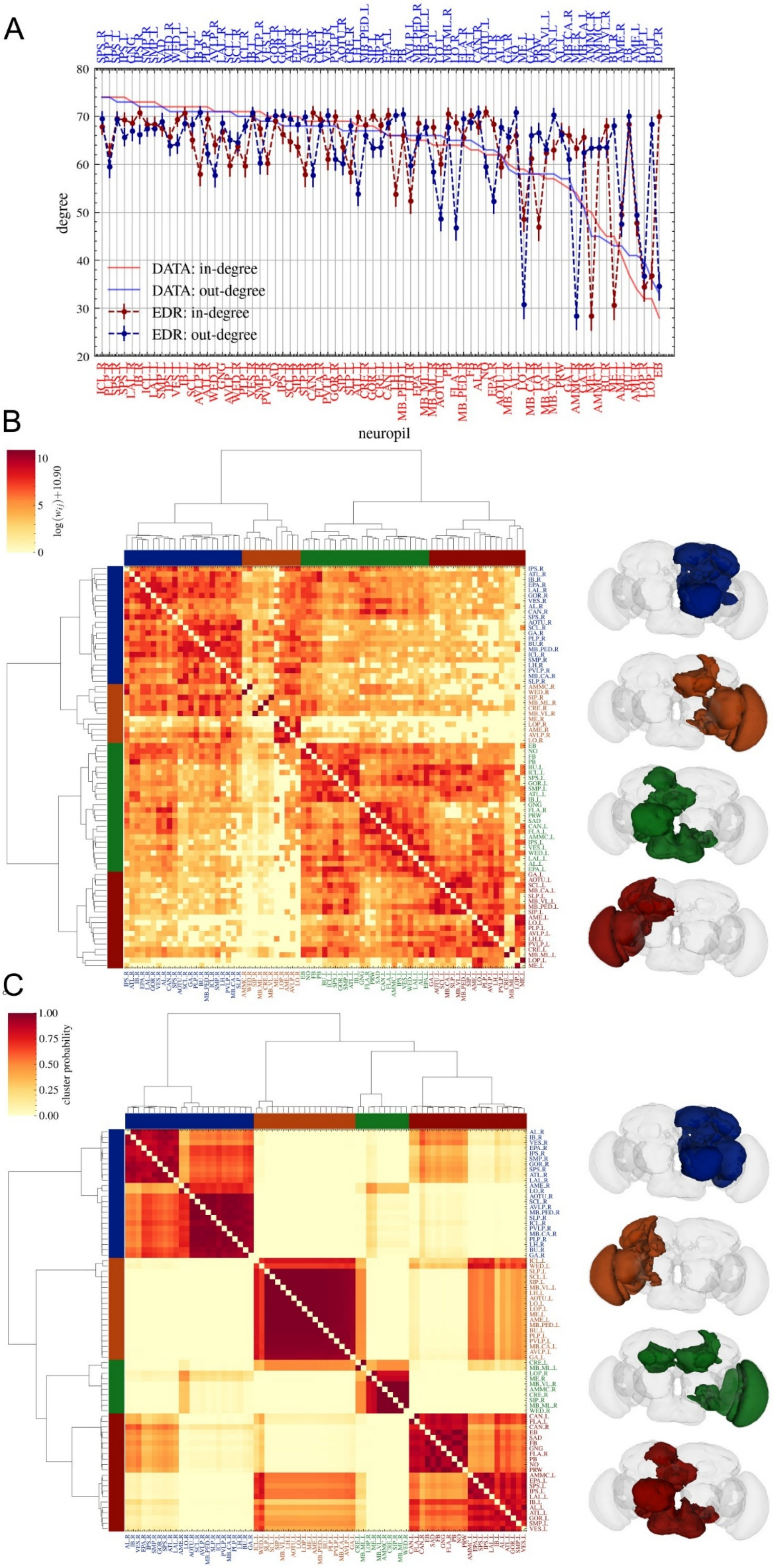

A
degree
DATA: in-degree
DATA: out-degree
EDR: in-degree
EDR: out-degree
neuropil
B
$\log(w_{ij})+10.90$
C
cluster probability

Supplementary Figure 7. Locally specific properties not reproduced by EDR model with $\lambda = 33\ mm^{-1}$. A) Properties specific to the nodes/neuropils (e.g. degree). Same sorting of the nodes is applied for both dataset and model results. The nodes with high degree are slightly underestimated by the model, nodes with middle degree values are relatively well estimated, and nodes with lowest degrees in the data have strongly fluctuating degrees in the model. B) Modular structure of one EDR model network. Hierarchical clustering is done using Ward's method, the 4 resulting clusters cut from the dendrogram are shown on the right. C) Determining modular structure of all EDR model networks. The same hierarchical clustering is performed for the ensemble of 1000 random networks, from which a contingency matrix is built representing the probabilities of the nodes being in the same cluster. This clustering is applied once again on this resulting matrix to get the groupings for all the model networks. The clusters are more localized in space and not symmetric.

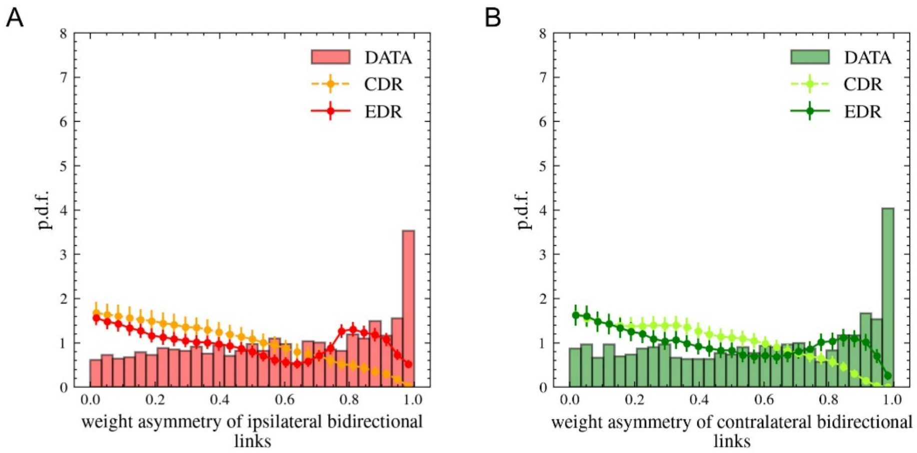

Supplementary Figure 8. Bidirectional weight asymmetry distribution. A) Weight asymmetry distribution for ipsilateral bidirectional links, and B) contralateral bidirectional links.

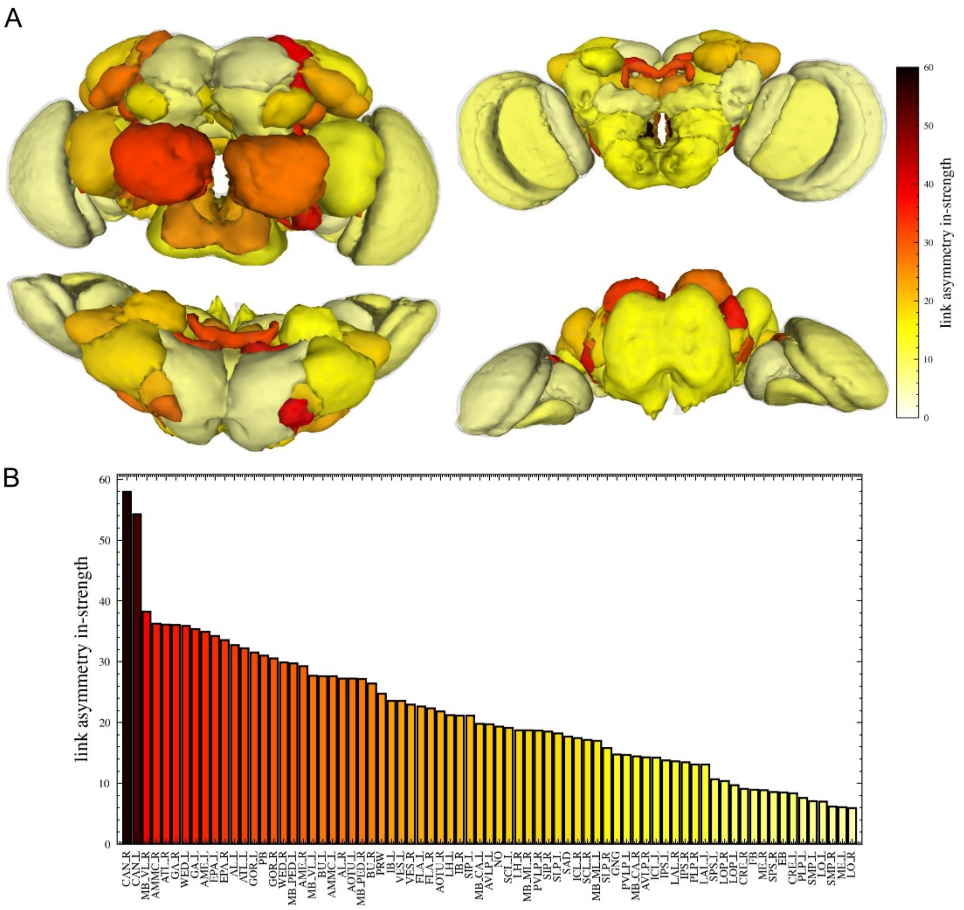

Supplementary Figure 9. **Brain map colored based on asymmetry in-strengths** A) Front, back, top and bottom view. B) Ranking of neuropils shown on bar plot.

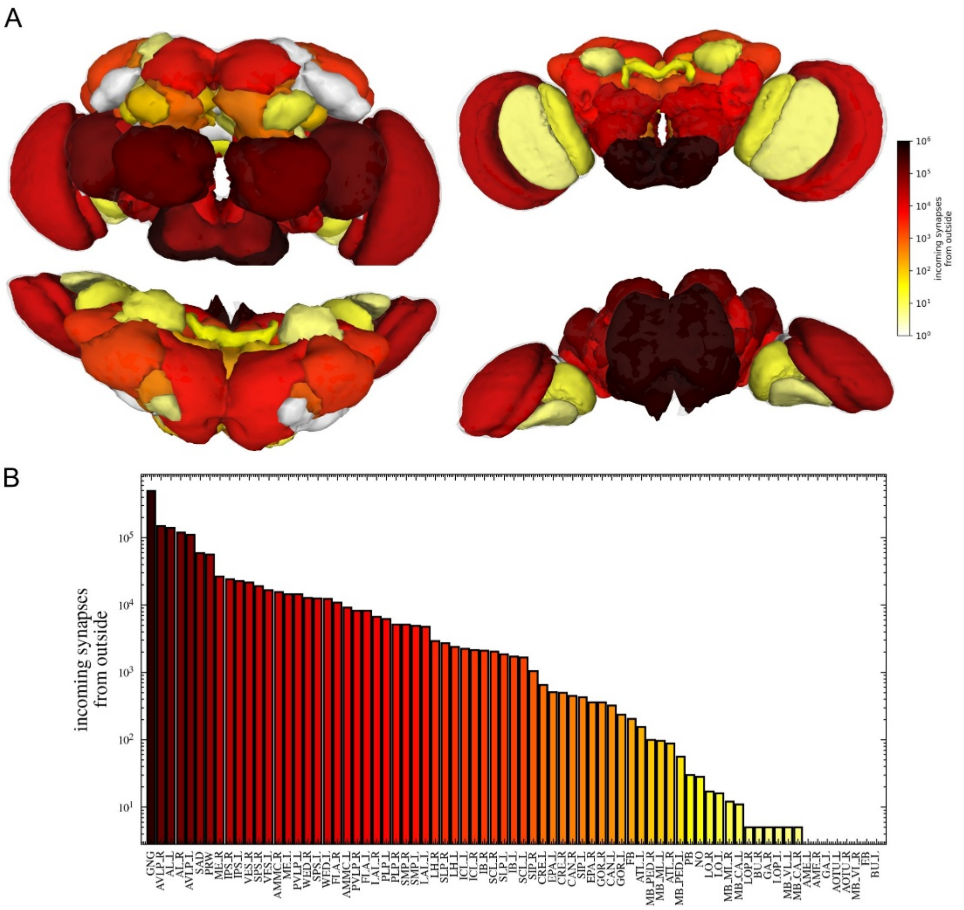

Supplementary Figure 10. Brain map colored based on incoming synapses from outside (presynapses of afferent neurons) A) Front, back, top and bottom view. B) Ranking of neuropils shown on bar plot.

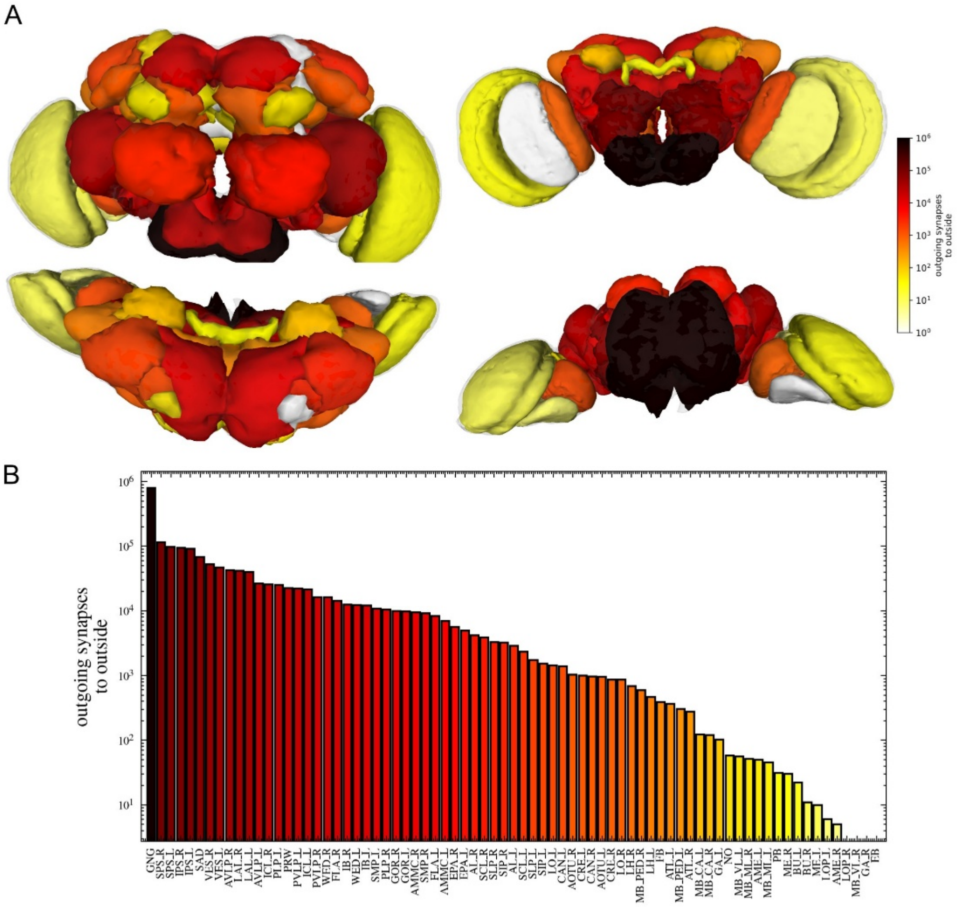

Supplementary Figure 11. Brain map colored based on outgoing synapses to outside (postsynapses of efferent neurons) A) Front, back, top and bottom view. B) Ranking of neuropils shown on bar plot.
